# Race-associated Molecular Changes in Gynecologic Malignancies

**DOI:** 10.1158/2767-9764.CRC-21-0018

**Published:** 2022-02-17

**Authors:** Amma Asare, Hui Yao, Olivia D. Lara, Ying Wang, Lin Zhang, Anil K. Sood

**Affiliations:** 1 Department of Obstetrics and Gynecology, Baylor College of Medicine, Houston, Texas.; 2 Department of Bioinformatics and Computational Biology, The University of Texas MD Anderson Cancer Center, Houston, Texas.; 3 Department of Obstetrics and Gynecology, NYU Langone Health, New York.; 4 Department of Obstetrics and Gynecology, University of Pennsylvania, Philadelphia, Pennsylvania.; 5 Center for RNA Interference and Non-Coding RNA, The University of Texas MD Anderson Cancer Center, Houston, Texas.; 6 Department of Gynecologic Oncology and Reproductive Medicine, The University of Texas MD Anderson Cancer Center, Houston, Texas.

## Abstract

The difference in cancer morbidity and mortality between individuals of different racial groups is complex. Health disparities provide a framework to explore potential connections between poor outcomes and individuals of different racial backgrounds. This study identifies genomic changes in African American patients with gynecologic malignancies, a population with well-established disparities in outcomes. Our data explore whether social health disparities might mediate interactions between the environment and tumor epigenomes and genomes that can be identified. Using The Cancer Genetic Ancestry Atlas, which encodes data from The Cancer Genome Atlas by ancestry and allows for systematic analyses of sequencing data by racial group, we performed large-scale, comparative analyses to identify novel targets with alterations in methylation, transcript, and miRNA expression between tumors from women of European American or African American racial groups across all gynecologic malignancies. We identify novel discrete genomic changes in these complex malignancies and suggest a framework for identifying novel therapeutic targets for future investigation.

**Significance:**

Common genetic changes in breast, ovarian, cervix, and uterine tumors can be identified in African American patients. Understanding why these changes occur may help improve outcomes for all patients with cancer.

## Introduction

Variations in morbidity, mortality, and treatment responses exist between individuals of different races and across multiple cancer types (1–3). In addition, differences in cancer incidence rates vary along racial and ethnic lines (4–6). However, even among patients who have cancer types with low incidence rates, non-whites have globally poorer outcomes, including higher rates of disease-related death than whites (7).

African American (AA) patients with gynecologic cancers are consistently found to have higher disease-associated mortality (8–11). Many studies highlight this inequity in patients with breast, ovarian, uterine, or cervical cancers, particularly when comparing AA patients to European American (EA) patients (12–17). Interactions among systemic and patient-, and provider-specific factors are known mediators of racial disparities in gynecologic malignancies (8–12). Identifying the role that social determinants of health, such as differential access to care, provider bias, and treatment location, play in disparities in gynecologic malignancies offers opportunities to develop targeted measures to promote equity.

High-throughput sequencing analyses of tumor samples are an important tool for identifying the molecular mechanisms of tumor initiation, progression, and metastasis (18–20). Malignancies of differing stages and severities harbor distinct alterations in messenger RNA (mRNA), miRNA, and DNA methylation. Genetic changes can be associated with subtypes of malignancies differing in severity, presentation, and mortality (21–23). These key genomic and epigenomic alterations are candidate targets for novel therapeutic drugs.

Historically, oncologic databases and clinical trials have not reflected the diversity of the United States population, underscoring the need for research centered on minorities (1, 17). Recent research efforts have used deep sequencing analyses to study tumors from individuals of different racial or ethnic backgrounds. Sequencing studies focusing on racial or ancestral differences can identify and characterize molecular changes in tumors from AA and EA patients (24, 25). However, interpreting these “race-associated” molecular changes in the context of health disparities and the socially constructed nature of race has proven complicated (4, 13, 26). Initiatives to address disparities in malignancies require a deeper characterization of the associated molecular changes to identify potential root causes. Crosstalk mediated by environmental factors is one proposed mechanism to explain the relationship between molecular changes and health disparities (25, 27–29). Thus, in this study, we aimed to characterize the molecular changes associated with racial disparities in breast and gynecologic cancers.

This study used The Cancer Genome Atlas (TCGA) database, which contains genomic sequencing data for many different tumor types, in tandem with The Cancer Genome Ancestry Atlas (TCGAA), which encodes TCGA data by ethnic ancestry and allows for systematic race-associated analyses of sequencing data previously not racially coded. Using these two databases, we performed large-scale, comparative, pan-cancer analyses of gynecologic cancers focusing on alterations that differed in tumors from individuals of AA and EA racial groups. First, we performed sequential examinations of mRNA, miRNA, and methylation changes in breast, uterine, cervical, and ovarian cancers to determine whether any race-associated changes existed. Next, we characterized the identified race-associated alterations and identified molecular connections among them. We identify coordinate and epigenetically driven genomic changes specific to gynecologic cancers which may be suggestive of environmental impacts on individuals of different racial groups. Our analysis supports a model whereby epigenetic and genetic changes may be contextualized within social determinants of health to understand disparities in gynecologic malignancies.

## Materials and Methods

### Assignment of Race Association

Racial tumor sample assignments previously identified by the TCGAA project were mapped to each tumor, and samples were allocated to AA or EA groups according to these TGCAA designations (25). Each tumor sample in the TCGA is assigned a designation according to the following methodology. Per the TCGAA project, genetic ancestry was estimated via computational analysis of the relationship between patient sample and reference populations of known ancestry. Self-identified race and ethnicity (SIRE) designations were assigned incompletely to the samples in the original TCGA database at the time of enrollment. For those samples with both a TCGAA and SIRE designation, the race and ancestry assigned were identical in 95.6% of cases (25). To allow utilization of all tumor samples in the database of interest, the TCGAA designation was used for all samples; however, given substantial overlap between SIRE and TCGAA designations, we use the term “race-associated” to represent tumor race assignments. This terminology identifies the association between race and ancestry while acknowledging how the classification likely encompasses race more broadly.

### Transcriptome and miRNA Analysis

Publicly available, processed, aggregated data generated via Illumina HiSeq gene expression profiling of ovarian, breast, cervical, uterine corpus endometrial cancer, and uterine carcinosarcoma tumors from TCGA were gathered using the FireHose data repository in accordance with the TCGA's data usage policy (RRID:SCR_003193). TCGA contains molecular, histopathologic, and limited clinical data for more than 11,000 patients with approximately 30 tumor types. RSEM normalized counts generated by the TCGA consortium analyses were used for downstream transcriptional analysis (30). Duplicated samples represented varying combinations of individual, sequencing platform, and data types. For our analysis based on individual tumor changes, only 1 sample per tumor type per individual was permitted. Analysis was performed within the R statistical computing software.

The method of differential expression analysis was performed as described previously (31). edgeR,RRID:SCR_012802 was used to transform data into a counts matrix with embedded genomic features and sample names. Low expression transcripts or miRNAs with less than 5 counts per million were excluded using filtration commands within edgeR. Libraries were normalized via the trimmed mean of M-values method and batch corrected. A Linear models for microarray analysis LIMMA,RRID:SCR_010943 pipeline was used to transform the data and estimate mean variance prior to linear modeling (31). The voom transformation was used and differential expression analysis was performed via limma with contrast matrix specifying comparison of AA and EA samples. For tumor-specific analyses, tumor type was specified in the contrast matrix and AA and EA samples and compared to identify differentially expressed genes.

Significantly altered mRNAs were considered transcripts with 2-fold changes (expressed in log_2_) and multiple hypothesis testing–adjusted *P* values (FDR) less than 0.05. Given the lower expression levels of miRNAs, significantly altered miRNAs were defined more permissively as those with 50% increased or decreased expression and multiple hypothesis testing–adjusted *P* values less than 0.05. Differentially expressed transcripts or miRNAs were identified for all gynecologic malignancies combined via a comparison of AA and EA ancestry samples.

### Methylation Analysis

The normalized and batch-effect–corrected beta values of 5 TCGA tumor types were generated using a probe-by-probe proportional rescaling method to yield a common set of probes with comparative methylation levels. This included methylation levels of 22601 CpG positions from 2,279 samples (367 AAs and 1912 EAs) of 981 breast invasive carcinoma (BRCA), 225 cervical squamous cell carcinoma and endocervical adenocarcinoma (CESC), 534 ovarian serous cystadenocarcinoma (OV), 486 uterine corpus endometrial carcinoma (UCEC) and 53 uterine carcinosarcoma (UCS). Illumina Infinium DNA methylation bead arrays, including both HumanMethylation27 (HM27) and Human Methylation450 (HM450) were incorporated. Briefly, difference between HM27 and HM450 by two distinct technical replicates was measured and a proportional rescaling method applied to remove platform effects. Please see original publication for full description (32). Individual probes were analyzed, no gene level aggregation was performed, and multiple probes mapping to the same gene were considered. The beta values were transformed to M-values. A linear model was fit with two covariates, ancestry type (EA vs. AA) and tumor types, per probe. *P* values were calculated using the moderated t-statistics. The Benjamini and Hochberg (BH) correction for multiple hypothesis testing was used to assess FDR. For probes with differential methylation of FDR < 0.05, the ∆ (AA – EA) of the beta values was calculated. The ∆ values for each probe were used to rank the probes for downstream gene-set enrichment analyses and are represented scaled and normalized in Fig. 3A. The significantly altered methylation probes were defined as those with a ∆ beta value greater than 0.1 and an adjusted *P* value less than 0.05.

### Pathway and Statistical Analysis

Functional pathway analysis was performed using Gene Set Enrichment Analysis (GSEA, Broad Institute and the University of California, SeqGSEA,RRID:SCR_005724) with the rank-based method for molecular profiling data and the Molecular Signatures Database v7.4. Significantly enriched pathways were those with a FDR (corrected for multiple hypothesis testing) *q* value of less than 0.05 and a normalized enrichment score or primary metric for scaling degree of enrichment of 2 or −2. Briefly, GSEA identifies biologically relevant groups of genes based on published or computationally predicted relationships. For example, the *MIR330* gene set includes genes with predicted binding sites for the miRNA *MIR330.* See publication for full description of GSEA methodology (33). For mRNA and miRNA GSEA analyses, FDR-adjusted *P* values were used to rank the transcripts. For methylation GSEA analyses, ∆ beta values and FDR-adjusted *P* values were used to rank the probes. No duplicated genes are allowed in the GSEA analysis and only the highest rank for duplicate genes was retained. MSigDB gene-set enrichment analysis was also used for methylation data (RRID:SCR_016863). All pathways displayed in the paper were significantly enriched (*q* < 0.05). We also used Ingenuity Pathway Analysis software (version 46901286; Ingenuity PathwayAnalysis, RRID:SCR_008653) to identify upstream regulation pathways with multiple hypothesis testing corrected (FDR) *P* values less than 0.05. The R environment was used to create all graphics and perform all associated statistical analyses. The *t* test was used to test against the null hypothesis of no difference between groups with the exception of Fig. 4C and Supplementary Fig. S5 and *P* values displayed. In Fig. 4C, the Spearman correlation coefficient was calculated for nonparametric data. The correlation line displayed was generated using a loess smoothing function. *χ*^2^ test comparing all probes to significantly altered probes was used for Supplementary Fig. S5. All figures were generated in the R environment using the ggplot2, RRID:SCR_014601 package. Heat maps were generated with R's pheatmap v1.0.12 RRID:SCR_016418 package using normalization followed by column-wise hierarchical Euclidean distance metric scaling.

### Comparison to Previously Identified Epigenetic Targets

Publications with social determinants of health-related epigenetic analysis were mined (34–40). All studies performed DNA methylation sequencing using Illumina DNA methylation bead arrays and identified specific probes as statistically significant in their own study. A total of 9,691 probes were identified. Common probes were identified.

### Data Availability Statement

The data generated in this study are available within the article and its Supplementary Data files.

## Results

### Transcriptional Alterations in Tumors from AA Patients

**FIGURE 1:**
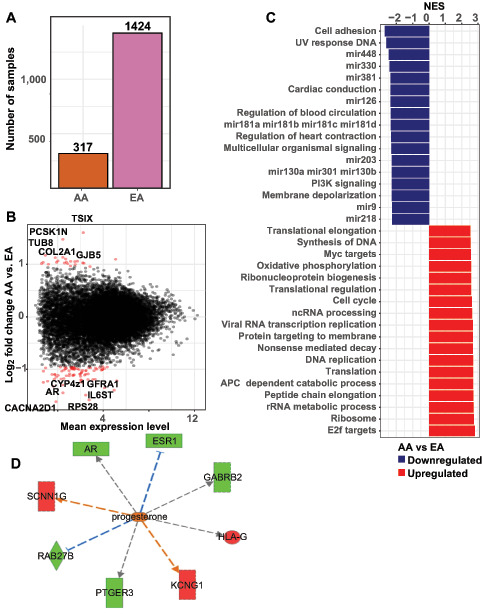
Transcriptional alterations in Pan-Gyn tumors from AA individuals. **A,** Number of ovarian, breast, cervical, and uterine tumor samples from AA and EA patients with mRNA sequencing included in The Cancer Genome Atlas (TCGA) consortium. **B,** Results of gene-set enrichment analysis of transcriptional changes in tumors from AA compared with EA patients. Significantly altered gene sets identified via Gene Set Enrichment Analysis with normalized enrichment scores (NES) are depicted. Gene sets up regulated in AA tumors are in red and gene sets down regulated in AA tumors are in blue. See Supplementary Table S2 for a complete list of significant genes set. **C,** Plot of identified transcript mean reads per kilobase of transcript per million mapped reads (RPKM) across all samples versus log transformed fold change in expression levels in AA versus EA (Log_2_ EA/AA). Significant transcripts were defined as those with two-fold expression change in AA versus EA samples and multiple hypothesis testing adjusted *P* values less than 0.05. Significant transcripts are highlighted in red. Names of the most-altered transcripts are noted on the plot. **D,** Ingenuity Pathway Analysis depiction of transcriptional changes in tumors from AA versus EA patients showing progesterone-associated changes in multiple transcripts. Relative changes in gene expression are depicted on a green (higher) to red (lower) scale for AA samples. Database-predicted activator relationships are orange; predicted inhibitor relationships are blue, and gray indicate unpredicted effects.

Previous work identified common changes across ovarian, cervical, uterine, and breast cancers, validating the utility of the study's “Pan-Gyn” cohort in genomic analyses (18). The role of race within the “Pan-Gyn” cohort, however, has not been explored. Here, TCGA samples from these four tumor types were sorted by racial group. Differential expression analysis performed on 1,741 tumor samples [81.8% EA (*n* = 1,424) and 18.2% AA (*n* = 317)] identified around 70 significantly altered transcripts when all tumors from AA and EA patients were combined and compared (Fig. 1A; Supplementary Fig. S1A; Supplementary Table S1). Differentially expressed transcripts were both up- and downregulated in tumors from AA compared with EA patients with a trend toward downregulation (Fig. 1B). Individual tumor-level analysis (breast, cervix, uterine, ovarian) of transcriptional changes between AA and EA racial groups identified distinct groups of differentially expressed transcripts (Supplementary Table S2). The number of significant transcripts varied greatly between individual tumor types. Significant transcripts were defined as transcripts with a *P* < 0.05 in multiple hypothesis testing–corrected differential expression analysis and 2-fold expression change between AA and EA tumors. While zero significantly different transcripts were identified in uterine carcinosarcoma, over 450 transcripts were significantly different between AA and EA tumors in the ovarian cancer samples. The absence of significant differences in the carcinosarcoma group and few differentially expressed targets discovered in the cervix and uterine groups reflect the low numbers of samples from AA patients available as well as tissue-specific variation.

The predominance of breast cancer samples within the “Pan-Gyn” cohort was reflected in the substantial overlap between racial group associated significantly differentially expressed genes in the breast tumor–specific analysis and the “Pan Gyn” analysis. Still, 21% of the “Pan-Gyn” identified transcripts were not identified in any other tumor subtype analysis. Despite the distinct organs of origin, the differing malignant potential of these cancer types, and the limited total numbers of AA samples, common changes could be found on the basis of patient race. This finding suggests that singular molecular alterations may occur in tumors from AA patients. Among the most significantly altered targets were multiple transcripts associated with the extracellular matrix and cell signaling such as *TUBB8, COL2A1*, and *CACNA2D1* (Fig. 1B).

Next, we performed pathway analysis to determine whether the transcript changes in AA versus EA tumors represented higher-order functional relationships (Fig. 1C; Supplementary Table S3). Upstream regulator pathway analysis in tumors from AA patients compared with EA patients showed decreased signaling in progestin-associated targets such as *AR,* the androgen receptor*,* consistent with the hormonally responsive breast and gynecologic tumors included in this cohort (Fig. 1D). We also identified decreased expression of *IL6ST*, IL6 cytokine family signal transducer, an inflammatory marker associated with response to the chemotherapeutic agent cisplatin (Supplementary Fig. S1B). Changes in these pathways may suggest a differential hormonal or treatment response in AA versus EA tumors. Decreased expression of PI3K signaling pathway molecules was seen in AA tumors, consistent with findings from analysis of AA samples in all TCGA tumors combined (25). Gene-set enrichment analysis identified other functional networks upregulated in AA tumors, including known transformation-related functions such as cell-cycle regulation and DNA synthesis. We also identified changes in pathways not previously associated with racial disparities or malignancy. For example, targets regulated by the transcriptional repressor *MEF2C* were significantly enriched in our analysis (Supplementary Fig. S1C). Pathway analysis was also performed on the ovary and breast tumor types individually as these two had the highest number of differentially expressed transcripts identified. Distinct candidate pathways were upregulated in ovarian tumors from AA patients including transcripts associated with microtubule assembly and ciliated motility (Supplementary Fig. S2A). Pathway analysis of breast tumors identified downregulation in miRNA-associated transcripts and activation in molecules involved in DNA replication and methylation (Supplementary Fig. S2B).

Next, pathways analysis was performed in the “Pan-Gyn” differentially expressed transcripts. The top differentially regulated pathways in the “Pan-Gyn” cohort notably included many miRNA-related gene sets composed of genes with common miRNA-binding sites. The binding sites for *MIR330*, *MIR381*, *MIR126*, and *MIR448* were the most under represented miRNA sites in AA tumors (Fig. 1B). While individual miRNAs, such as *MIR448*, have been previously associated with malignancy, the broader role of miRNA alteration in AA tumors is unknown (41). The abundance of many miRNA-associated pathway changes led us to directly explore differences in miRNA expression in AA tumors.

### miRNA Expression Changes in AA Tumors

**FIGURE 2:**
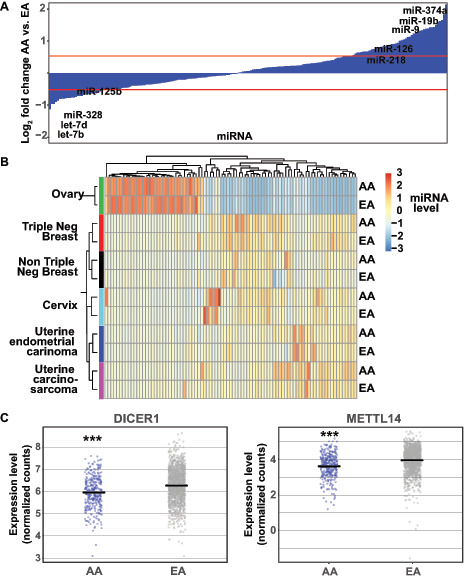
miRNA expression changes in tumors from AA individuals. **A,** Log_2_ fold changes in expression levels for all miRNAs. Red lines mark log_2_ changes greater than 0.5 or less than 0.5 with multiple hypothesis testing adjusted *P* values less than 0.05. Names of significant miRNAs previously associated with malignancy are shown. **B,** Heat map of normalized and scaled expression levels of the most differentially expressed miRNAs across ovarian, triple-negative breast, non-triple-negative breast, cervical, and uterine cancers. Vertical and horizontal dendrograms depict relationships between samples and miRNAs, respectively. **C,** Plots of relative mRNA expression levels (RPKM) in individual tumor samples for the miRNA processing proteins DICER1 and METTL14 in AA and EA tumors. Each dot represents a single tumor sample. Horizontal black lines indicate mean transcript expression level. Triple asterisks indicate *t* test adjusted *P* value less than 0.001.

To further investigate potential miRNA landscape changes, we compared the available miRNA sequencing profiles for 1402 EA tumors to those for 305 AA tumors. Differential expression analysis identified around 80 significant miRNA expression level changes between Pan-Gyn AA and EA tumors. Some of the miRNAs identified had lower expression levels in AA tumors than in EA tumors (Fig. 2A; Supplementary Table S4). The majority (77%) of the statistically significantly differentially expressed miRNAs were upregulated in AA tumors. As was noted in the transcriptional pathway analysis, a number of the identified miRNAs—including *MIR374A* and *MIR19B1*, the two most upregulated miRNAs—have previously been associated with malignancy (42–44).

Next, we compared tumor-specific miRNA expression changes. Hierarchical clustering grouped each tumor type, regardless of sample racial group, indicating the importance of tumor site in determining miRNA expression patterns (Fig. 2B). Previous work focused on the role of differential miRNA expression in the triple-negative breast cancer (TNBC) histologic subtype (estrogen receptor–negative, progesterone receptor–negative, HER2-negative (45, 46). TNBC subtype is also associated with increased morbidity and AA race (13). In addition, the TNBC subtype was identified within the TCGA sample–associated information and a modest number of samples were present to facilitate analysis (31 AA and 52 EA TNBC; 130 AA and 545 EA non-TNBC). Given this, we examined miRNA patterns in TNBC and non-TNBC separately. The TNBC samples clustered separately from the non-TNBC samples, however, were closely related. Compared with the breast, uterine, and cervical cancer samples, the ovarian cancer samples were most distinct in terms of miRNA expression. The changes in miRNA levels in breast and gynecologic tumors from AA patients raised the possibility of global dysregulation in miRNA synthesis, processing, or degradation in AA tumors. To investigate this hypothesis, we investigated the mRNA expression levels of known miRNA processing and modulation proteins using our transcriptome data (Fig. 2C). We found that, in the AA tumors, the mRNA expression levels of many core miRNA processing proteins were modestly, but significantly, decreased (Supplementary Fig. S3). Both the *DICER1* and *METTL14* miRNA processing proteins were transcriptionally downregulated in the AA tumors, suggesting a molecular etiology for these large-scale changes in miRNA levels (Fig. 2C).

### Epigenetic Alterations in AA Tumors

**FIGURE 3:**
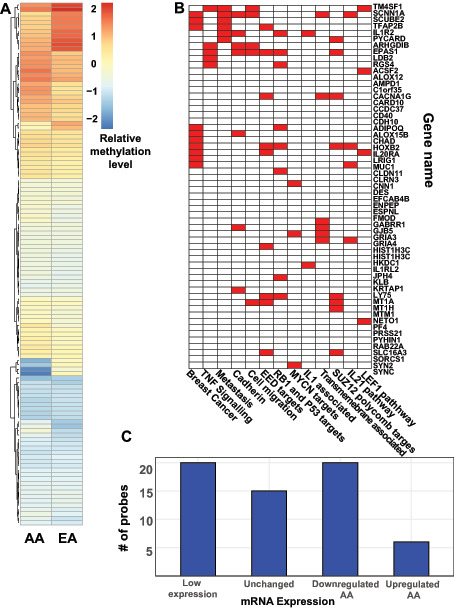
Epigenetic alterations in tumors from AA patients. **A,** Heat map of normalized and scaled methylation beta levels for 61 significantly altered probes identified in Pan-Gyn analysis. Probes with higher relative methylation are in red and those with lower relative methylation are in blue. **B,** Gene-set enrichment analysis of 61 significantly altered methylation probes. Gene set names are shown on the horizontal axis and genes are shown along the vertical axis. The red-filled boxes mark gene membership within each gene set. **C,** Bar plots of association between 61 significantly altered probes and mRNA expression in AA tumors at the corresponding gene. Genes were either upregulated, downregulated, unchanged or lowly expressed in AA tumors relative to EA tumors. The number of mRNA expression changes associated with probes with significant changes in DNA methylation are counted on the *y*-axis.

Global epigenetic changes are associated with transformation in many types of malignancies (47–51). Changes in both types of epigenetic regulation mechanisms, DNA methylation, and chromatin modification, are seen in gynecologic malignancies (52, 53). To investigate whether epigenetic alterations in gynecologic cancers are associated with patient racial group, we searched for common changes in AA and EA tumor samples. Using the DNA methylation data in the TCGA database, we compared the extent of methylation and its locations in AA and EA tumors. When comparing EA tumors and AA tumors, differential methylation analysis identified many alterations, including 61 probes that were significantly altered (Fig. 3A; Supplementary Table S5). These data suggest that methylation marks are distributed differently in AA tumors. The differentially methylated genes were distinct from those identified by comparing AA and EA tumors within any single cancer type (Supplementary Fig. S4). To investigate whether common AA tumor–associated methylation patterns were associated with functional changes in cell behavior, we performed gene-set enrichment analysis on the subset of significantly altered genes and found a number of common pathways among our changes (Fig. 3B). Gene sets associated with malignant features such as metastasis, cadherin signaling, and cell migration were enriched among significantly differentially methylated probe-associated genes. When we looked more closely at the changes in methylation at each locus, we found that 21% had decreased methylation in AA tumors and the remaining majority had increased methylation in AA tumors, suggesting both a global difference in methylation quantity and differences in location. Analysis of methylation patterns in each tumor type was also performed (Supplementary Fig. S4; Supplementary Table S6). Similar to the transcriptional analysis, differing quantities of significant probes were identified in each tumor type. When comparing the significantly altered probes in breast to the Pan-Gyn cohort 52% of the probes identified in the Pan-Gyn analysis were unique. We then returned to the Pan-Gyn cohort to better understand how these methylation changes could affect cell function. We further determined which of our significantly altered genes could be correlated with gene expression changes in our transcriptome analysis.

Although DNA methylation of a gene is most commonly associated with the repression of gene expression, the expression levels of a hypermethylated gene may increase or decrease. Thus, we examined whether identified genes with significantly altered DNA methylation also had significant changes in mRNA expression (Fig. 3C). While some genes harboring methylated loci of interest were not expressed in AA or EA samples, approximately half of the loci identified in the methylation analysis had significant mRNA expression changes. After methylation, downregulation of expression was more common than upregulation, as expected.

**FIGURE 4:**
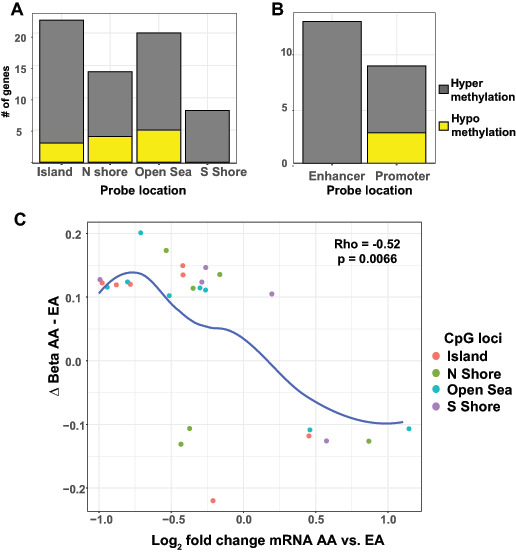
Association between methylation and mRNA alterations. **A,** Bar plots of differentially methylated probes categorized by loci relative to CpG islands. The number of probes hypermethylated in the AA samples is shown in gray and the probes hypomethylated in the AA samples are depicted with yellow bars. **B,** Bar plots of differentially methylated probes assigned to enhancer or promoter regions. The number of probes hypermethylated in AA samples is shown in gray and the probes hypomethylated in the AA samples are depicted with yellow bars. **C,** Scatter plot of the change in beta methylation levels in AA versus EA samples and the log_2_ change in mRNA expression. The location of each probe relative to a CpG island is indicated by color. The blue line highlights statistically significant correlations between changes in methylation expression levels and changes in mRNA levels as assessed by the Spearman rank-order correlation.

The effect of DNA methylation on gene expression is mediated by the location of the methylation mark in the gene structure. Accordingly, we next evaluated where methylation changes were located within DNA, relative to the nearest gene. Methylation changes are often characterized in terms of their relationships to CpG dinucleotides. Clusters of CpG dinucleotides exist in highly repetitive promoter-associated DNA regions known as CpG islands. Methylation changes at CpG islands consistently alter gene expression in normal and malignant cells. We found that significant methylation alterations were distributed throughout the genes of interest, with 30% located within CpG islands (Fig. 4A). With respect to other structural landmarks, our identified methylation changes were distributed across the gene structure with an increased proportion in the 5′ UTR and TSS1500 (promoter) regions (Supplementary Fig. S5). Most of the significantly altered loci were hypermethylated in AA tumors relative to EA tumors and included changes at enhancer regions (Fig. 4B and C). These patterns suggest that the identified methylation changes were potential mediators of gene expression and subsequent cellular function. To further characterize the relationship between methylation changes and gene expression, the correlation between the degree of methylation alteration and the change in mRNA expression was plotted (Fig. 4C). Notably, larger changes in methylation between AA and EA tumors correlated significantly with larger changes in mRNA expression.

## Discussion

In breast and gynecologic malignancies, disparities among individuals of different racial backgrounds are pervasive and the subject of recent inquiries (1, 17, 54). The paucity of samples from AA patients within the TCGA database highlights the need to include patients of color in research studies. Although genomic analysis has previously been used to characterize racial differences, we applied it specifically to malignancies of the breast, uterus, cervix, and ovary. Our first goal was to understand whether a sequencing-based genomic approach could be used to characterize molecular changes potentially associated with the known outcome disparities between AA and EA individuals. This multidimensional analysis of genomic data identified novel changes in mRNA expression, miRNA expression, and DNA methylation in breast and gynecologic cancers from patients of AA and EA racial groups. Despite the distinct cells of origin of these tumors, the unique tissue types, and the natural history of each malignancy, race was sufficient to identify common genomic changes. We also examined tumor-specific transcriptional and DNA methylation changes in AA and EA patients and have created a database of transcripts and probes correlated with racial groups for candidate and validation approaches in the future.

This study defined a novel set of candidate transcripts that could be involved in the higher morbidity and mortality of breast and gynecologic cancers in AA patients. We identified transcriptome alterations in AA tumors consistent with malignant progression and metastasis, such as increased DNA synthesis, increased E2F target expression, and decreased cell-adhesion molecule expression. We also found coordinated changes in pathways less studied in malignancy, including those for noncoding RNA processing, ribosome-associated factors, and RNA metabolism, suggesting areas of focus for ongoing research (Supplementary Table S2). Individual transcripts with the most significant changes in AA patients included *AR* and *GFRA1*, which have been previously implicated in Pan-Gyn malignancies and breast cancer, respectively (18, 55). This suggests that other significantly altered transcripts such as *RPS28, CYP4Z1*, *and IL6ST* may represent novel biomarkers or candidate regulators of tumor progression (Supplementary Table S1).

In this study, we examined whether the observed transcriptional changes represented alterations in larger functional networks or cell behaviors. Transcriptome-wide gene-set enrichment analysis identified multiple downregulated, miRNA-associated gene networks. We discovered a concomitant, global upregulation of miRNA expression in AA tumors. These findings suggest that elevated levels of miRNAs in AA tumors may mediate systems of transcriptional repression and subsequent functional differences in tumor biology. This shift in the miRNA landscape raised the possibility of global changes in miRNA biogenesis or processing in AA tumors. Indeed, we identified decreased expression of miRNA and mRNA biogenesis transcripts such as *DICER1* and *METTL14* in AA tumors. Although one might expect reduced levels of *DICER1*, a protein essential for production of miRNA, to correlate with reduced levels of miRNA, our data showed increased expression of select miRNAs. Given that changes in global miRNA abundance are not easily assessed in this data set, this shift in miRNA expression may be consistent with a reduction in total miRNA levels. Alternatively, this change may suggest negative regulation of *DICER1* transcription secondary to elevated individual miRNA levels or other secondary means of regulating miRNA quantity. This complex regulation network warrants further dedicated study. Our parallel identification of transcriptional and miRNA changes allows a better understanding of molecular differences between tumors from AA and EA individuals.

The addition of methylation analysis further enriched our understanding of how AA and EA tumors are molecularly distinct. In the AA tumors, we identified common and significant methylation changes that were distinct from those seen upon analysis of each cancer type individually. Of note, the genes associated with the significant methylation changes were enriched in functional clusters previously implicated in our analysis (e.g., cadherin-mediated cell adhesion) as well as in other pathways of interest, such as the Wnt signaling pathway, and the pathway associated with the histone-modifying polycomb molecules *EED* and *SUZ12*. The changes in the methylation of polycomb protein-regulated targets suggest cross talk with histone modification, another important mechanism of epigenetic control. Importantly, the methylation changes we identified were correlated with alterations in gene expression, underscoring the functional significance of the alterations. Our model suggested interdependence among the genomic landscape shifts we identified. This underscores the importance of clearly defining these relationships when assessing candidacy for therapeutic targeting.

We defined multiple, interconnected genomic landscape, and individual locus changes correlated with racial groups in breast and gynecologic malignancies. Our study and other large-scale genomic analyses based on race support the hypothesis that epigenetic alterations may be important mediators of genomic differences. Given these and other data identifying epigenetic differences between racial groups, elucidating a link between social determinants of health, environmental factors, and downstream genome alterations is an important mission for future study (24, 25, 56). Epigenetic changes can be mediated by environmental factors such as developmental context, health care access, food sources, and toxin exposure. Each of these environmental factors is inseparable from the social determinants of health defined by an individual's racial identity. Rather than supporting the causal role of any single germline genetic change, these data support an integrated approach to the genomic analysis of racial disparities in cancer focused on identifying key epigenetic changes.

To explore this possibility more closely, we evaluated our identified significant methylation probes for known associations with social determinants of health factors. Both probes identified from tumor-specific methylation analysis and “Pan-Gyn” methylation analysis were evaluated. Published studies identifying methylations probes associated with smoking, maternal toxin exposure, and dietary alterations were mined and a library of probes was created (34–40). The 11 probes identified both in this study as well as other epigenetic studies are listed in Supplementary Table S7. Interestingly, 10 of the 11 probes were identified as differentially methylated after exposure to smoking. Two probes identified by the “Pan-Gyn” methylation analysis were also identified via this analysis. Probes mapping to *TM4SF1* and *SYN2* were identified as potential candidate environment–genetic mediators and significantly altered in the “Pan-Gyn” cohort (37, 40). Yet only a minority of the total significant probes identified in this study were previously modified by known environmental stressors. This minimal overlap suggests that external triggers of epigenetic changes, if present, may be diverse. Such triggers may range from environmental mutagens such as cigarette smoking to stress-related factors such as PTSD.

Our study is notably restricted by limited numbers of samples from AA patients and limited clinical information about all patients. Factors such as smoking status, BMI, comorbid conditions, or place of residence cannot be assessed in our data set. Without this information, causal links between clinical outcomes and social determinants of health cannot be specifically identified. In addition, as with the majority of pooled sequencing studies, the skewing contributions of different types of cells within the tumor or tumor heterogeneity cannot be parsed.

While this particular study cannot directly evaluate interactions between individual outcomes, race, and the environment, considering potential links between social determinants of health and tumor biology represents a new framework for therapeutic focused translational research.

## Supplementary Material

Supplemental Table 1 Differentially expressed transcripts in Pan-Gyn cohort EA vs AA

Supplemental Table 2 Differentially expressed transcripts in individual tumor types

Supplemental Table 3 Gene Set Enrichment Analysis of transcriptional changes in AA samples

Supplemental Table 4 Differentially expressed microRNAs in Pan-Gyn cohort EA vs AA

Supplemental Table 5 Differentially methylated probes in Pan-Gyn cohort

Supplemental Table 6 Differentially methylated probes analyzed by tumor site

Supplemental Table 7 Differentially methylated probes identified independently in epigenetic studies of environment

Supplemental Figure S1 Detailed patient racial assignments by tumor type and additional pathway analysis

Supplemental Figure S2 Analysis of mRNA expression by tumor type

Supplemental Figure S3 Expression levels of RNA-processing pathway members

Supplemental Figure S4 Analysis of methylation changes by tumor type

Supplemental Figure S5 Analysis of differentially methylated probe locations
