# Supplementary material for: Race-associated Molecular Changes in Gynecologic Malignancies
Source: Cancer Res Commun. 2022 Feb 17;2(2):99–109. doi: 10.1158/2767-9764.CRC-21-0018 (PMC9390975; doi:10.1158/2767-9764.CRC-21-0018)
Supplement: Supplemental Table 1 — Differentially expressed transcripts in Pan-Gyn cohort EA vs AA [file crc-21-0018-s01.pdf]

Supplemental Table 1

Gene	logFC EA/AA	adj.P.Val
CACNA2D1	-1.616486248	2.73234E-15
RPS28	-1.583192857	7.98044E-10
AR	-1.490650597	2.38261E-09
CYP4Z1	-1.447292302	1.2563E-06
IL6ST	-1.39709581	5.56145E-12
GFRA1	-1.350601496	0.00012253
KCND3	-1.319183545	2.55279E-12
AGTR1	-1.295185468	1.00719E-07
PRKG2	-1.247744271	1.00429E-18
ADAMTS15	-1.243349701	1.10456E-09
C7	-1.2367314	9.23632E-06
IGSF9B	-1.22560262	1.98458E-11
SLC7A2	-1.219174304	2.19199E-08
GPR81	-1.205194981	9.03106E-08
EDIL3	-1.196038236	2.67603E-11
CYP4B1	-1.191310314	1.0731E-06
CLIC6	-1.1889956	1.53997E-07
ERBB4	-1.185121909	1.38167E-05
TIMP4	-1.184578067	3.87282E-08
THRSP	-1.180747663	7.73563E-06

NRK	-1.172764212	3.89521E-10
GABRB3	-1.146063066	1.83977E-08
FMO2	-1.131628919	2.03438E-06
OMD	-1.122322689	1.03144E-06
ARHGAP20	-1.121317355	4.99001E-16
CYP2B7P1	-1.118740042	0.000325526
CDKL5	-1.114267352	3.01303E-15
CCNT1	-1.108688716	4.51526E-20
CNTN1	-1.106268555	8.30467E-10
PTPRT	-1.104947401	0.00043836
SLC28A3	-1.097832524	3.16207E-07
RUNX1T1	-1.097034394	3.07801E-12
FAT3	-1.096850844	1.34563E-11
ZFPM2	-1.092742421	2.06064E-14
CHL1	-1.09142626	1.49816E-07
RASEF	-1.086500505	2.37049E-09
NFATC2	-1.086065811	1.01759E-12
OGN	-1.082412447	1.42436E-05
KIAA0754	-1.06592747	3.87592E-14
ADAMTS12	-1.045849428	3.13298E-09
KLHL4	-1.03585655	1.98589E-12
PRKG1	-1.034774571	1.12459E-10
SLC44A5	-1.026319148	7.14098E-08

DDR2	-1.02610626	2.20563E-11
KIRREL	-1.025751468	1.71401E-11
LRRC37A2	-1.02131282	2.3834E-42
TSHZ2	-1.017308713	2.93245E-13
COL10A1	-1.014043685	0.00018806
TNN	-1.014001509	3.27188E-06
AKAP2	-1.004992219	3.01423E-10
GRIK3	-1.002663592	1.41135E-05
FMO1	-1.002428093	1.04516E-09
GGT3P	1.002995458	5.209E-17
KCNG1	1.012630355	3.03523E-08
RNF112	1.015831752	1.4871E-25
P2RY11	1.022090961	5.32665E-13
GPC2	1.025637556	1.47321E-17
MSLN	1.030129161	0.001066345
INE2	1.038603464	4.36059E-12
CCL3L1	1.038777254	4.62231E-18
TSPAN10	1.044760145	2.40626E-24
NACA2	1.053746115	1.67191E-30
COL9A3	1.058009115	3.07866E-11
MAGED4	1.061320144	6.47747E-14
MFSD2B	1.065604315	3.40299E-19
CAPS	1.097430224	8.36083E-17
DLX5	1.10407257	2.02685E-

		10
GJB5	1.1274652	7.11978E-09
COL2A1	1.135461778	5.03194E-06
TUBB8	1.173527118	1.83113E-35
PCSK1N	1.477253295	3.12726E-12
TSIX	1.605997143	2.74797E-22

Gene: gene name

LogFC AA vs EA; Log2 (mean EA tumor RPKM) / (mean AA tumor RPKM) for each transcript

adj.P.Val: P value adjusted for multiple hypothesis testing with BH method
