# Supplementary material for: Race-associated Molecular Changes in Gynecologic Malignancies
Source: Cancer Res Commun. 2022 Feb 17;2(2):99–109. doi: 10.1158/2767-9764.CRC-21-0018 (PMC9390975; doi:10.1158/2767-9764.CRC-21-0018)
Supplement: Supplemental Table 2 — Differentially expressed transcripts in individual tumor types [file crc-21-0018-s02.pdf]

Supplemental Table 2

Gene	logFC	adj.P.Val	Tumor
GSTM1	3.54	1.07E-03	CESC
HBB	1.78	4.65E-02	CESC
GSTT2	1.22	4.65E-02	CESC
IHH	2.46	3.86E-02	UCEC
PDIA2	1.63	4.82E-02	UCEC
HSD17B3	1.51	6.23E-03	UCEC
GPR83	1.37	1.37E-02	UCEC
LRGUK	1.22	3.36E-02	UCEC
C3orf32	1.17	1.58E-02	UCEC
NOTCH2NL	1.14	2.54E-10	UCEC
LRRIQ3	1.03	4.64E-02	UCEC
DISP2	-1.16	5.88E-03	UCEC
C21orf56	-1.30	3.80E-02	UCEC
RPS28	-2.49	8.28E-03	UCEC
ULK4	1.18	7.82E-08	OV
PLCH1	2.03	2.42E-07	OV
ACSM3	1.34	1.53E-05	OV
CEACAM21	-2.03	1.53E-05	OV
CD300C	-1.81	4.05E-05	OV
EFR3B	1.34	4.40E-05	OV
C1QC	-1.51	4.93E-05	OV
SLC26A7	1.99	4.93E-05	OV
BSN	1.28	5.09E-05	OV
ARHGDIB	-1.15	5.09E-05	OV
C1QB	-1.55	7.39E-05	OV
TYROBP	-1.41	9.70E-05	OV
C3orf15	1.20	1.03E-04	OV
FGFRL1	1.21	1.47E-04	OV
GPSM3	-1.33	1.54E-04	OV
C1QA	-1.45	1.71E-04	OV
TBX1	1.85	2.05E-04	OV
ZNF389	1.22	2.24E-04	OV
PDLIM2	-1.02	2.60E-04	OV
GYPC	-1.26	2.77E-04	OV
LAPTM5	-1.31	2.90E-04	OV
TGFBI	-1.23	3.84E-04	OV
HLA-DPA1	-1.51	4.42E-04	OV
FILIP1	1.37	4.44E-04	OV

CRYBB1	-1.38	5.03E-04	OV
HNMT	-1.10	5.03E-04	OV
HS3ST1	-1.21	5.03E-04	OV
CD68	-1.12	5.27E-04	OV
MDH1B	1.01	5.27E-04	OV
SRGN	-1.15	5.59E-04	OV
MSI1	1.57	5.73E-04	OV
SIGLEC9	-1.49	6.33E-04	OV
SLCO2B1	-1.42	6.33E-04	OV
AIF1	-1.52	6.34E-04	OV
FCER1G	-1.28	6.44E-04	OV
C2orf39	2.04	6.91E-04	OV
ZNF334	1.19	6.93E-04	OV
ZNF443	1.04	6.93E-04	OV
CRIP3	1.49	7.19E-04	OV
DNAH7	1.25	7.19E-04	OV
DNAH10	1.27	7.37E-04	OV
SLC46A3	-1.04	8.02E-04	OV
HYDIN	1.13	8.52E-04	OV
MAF	-1.20	8.58E-04	OV
DNAH6	1.45	8.87E-04	OV
GLRX	-1.00	8.87E-04	OV
MS4A4A	-1.50	8.90E-04	OV
LOC100233209	-1.69	9.43E-04	OV
CD14	-1.18	9.93E-04	OV
C17orf60	-1.56	1.00E-03	OV
LRRC37A2	-1.17	1.00E-03	OV
GIMAP6	-1.03	1.27E-03	OV
ADORA3	-1.52	1.29E-03	OV
CYBB	-1.51	1.35E-03	OV
LSP1	-1.15	1.35E-03	OV
HAMP	-1.81	1.35E-03	OV
RNASE2	-1.50	1.38E-03	OV
CLEC18A	1.57	1.48E-03	OV
NPL	-1.03	1.49E-03	OV
C1orf162	-1.20	1.50E-03	OV
RNASE6	-1.41	1.53E-03	OV
C20orf26	1.56	1.58E-03	OV
LILRA2	-1.45	1.58E-03	OV
LRP4	1.44	1.58E-03	OV
MS4A6A	-1.50	1.58E-03	OV

RCSD1	-1.05	1.58E-03	OV
DCN	-1.49	1.61E-03	OV
TBXAS1	-1.31	1.68E-03	OV
LRRIQ3	1.03	1.71E-03	OV
CACNB4	1.04	1.78E-03	OV
CCDC74B	1.02	1.82E-03	OV
MGC16121	-1.59	1.82E-03	OV
ADAP2	-1.01	1.84E-03	OV
GNG2	-1.01	1.85E-03	OV
HLA-DPB1	-1.33	1.85E-03	OV
GMFG	-1.07	1.91E-03	OV
APOBEC3H	-1.44	1.94E-03	OV
DSCAML1	1.63	2.06E-03	OV
GIMAP4	-1.11	2.06E-03	OV
MSR1	-1.24	2.07E-03	OV
FOLR2	-1.25	2.07E-03	OV
PIK3CG	-1.39	2.07E-03	OV
CD300A	-1.19	2.12E-03	OV
IRF8	-1.44	2.12E-03	OV
PDIA2	1.82	2.19E-03	OV
APOC2	-2.01	2.24E-03	OV
C1S	-1.18	2.24E-03	OV
FCGR2A	-1.14	2.24E-03	OV
GAB3	-1.12	2.24E-03	OV
ABLIM3	-1.11	2.24E-03	OV
EFEMP1	-1.89	2.28E-03	OV
ITGB2	-1.24	2.28E-03	OV
DNAH11	1.60	2.39E-03	OV
CD52	-1.56	2.40E-03	OV
LAIR1	-1.36	2.57E-03	OV
SELPLG	-1.16	2.66E-03	OV
S1PR4	-1.20	2.72E-03	OV
LY86	-1.29	2.72E-03	OV
MT2A	-1.06	2.75E-03	OV
HKDC1	1.23	2.75E-03	OV
CRABP2	-1.16	2.76E-03	OV
RSPH10B2	1.35	2.77E-03	OV
CSF1R	-1.22	2.77E-03	OV
VSIG4	-1.43	2.81E-03	OV
ALOX5AP	-1.69	2.85E-03	OV
SIGLEC7	-1.18	2.87E-03	OV

PPARG	-1.34	2.89E-03	OV
HAVCR2	-1.32	2.90E-03	OV
HOPX	-1.29	2.95E-03	OV
RNF157	1.08	2.95E-03	OV
GIMAP1	-1.00	3.02E-03	OV
TTLL9	1.19	3.03E-03	OV
GPR34	-1.39	3.07E-03	OV
F13A1	-1.52	3.10E-03	OV
WDR65	1.46	3.29E-03	OV
CLEC18B	1.53	3.32E-03	OV
MS4A7	-1.15	3.39E-03	OV
TMEM176A	-1.54	3.59E-03	OV
CMKLR1	-1.17	3.66E-03	OV
HLA-DMA	-1.18	3.66E-03	OV
HCST	-1.06	3.73E-03	OV
LRRC25	-1.12	3.82E-03	OV
LY96	-1.22	3.87E-03	OV
CTSW	-1.90	3.93E-03	OV
JAK3	-1.01	3.93E-03	OV
SIRPB2	-1.44	3.93E-03	OV
SLC38A3	1.34	3.93E-03	OV
GAL3ST4	-1.01	4.01E-03	OV
GNG7	-1.35	4.01E-03	OV
CD300LF	-1.41	4.04E-03	OV
CXCL12	-1.65	4.15E-03	OV
CD163	-1.34	4.20E-03	OV
SIGLEC11	-1.84	4.20E-03	OV
TNFSF8	-1.48	4.22E-03	OV
FCGR3A	-1.24	4.26E-03	OV
PSTPIP1	-1.20	4.42E-03	OV
TUBB2B	2.14	4.42E-03	OV
ARHGAP9	-1.31	4.43E-03	OV
FERMT3	-1.09	4.48E-03	OV
CD37	-1.12	4.55E-03	OV
CSF3R	-1.54	4.55E-03	OV
PRDM8	-1.19	4.58E-03	OV
CSF2RA	-1.44	4.58E-03	OV
KLRB1	-2.02	4.58E-03	OV
PIK3R5	-1.05	4.64E-03	OV
RASGRP4	-1.19	4.64E-03	OV
LST1	-1.37	4.73E-03	OV

HIST1H4C	-1.12	4.91E-03	OV
SPI1	-1.07	4.91E-03	OV
BTK	-1.35	5.19E-03	OV
WAS	-1.03	5.20E-03	OV
CYTH4	-1.17	5.29E-03	OV
CA14	1.10	5.38E-03	OV
ABI3	-1.05	5.39E-03	OV
LYZ	-1.56	5.39E-03	OV
GIMAP7	-1.02	5.47E-03	OV
TLR4	-1.07	5.49E-03	OV
P2RY13	-1.47	5.57E-03	OV
DOCK8	-1.12	5.62E-03	OV
CD86	-1.27	5.72E-03	OV
LAT2	-1.16	5.80E-03	OV
VENTX	-1.25	5.83E-03	OV
SIGLEC5	-1.29	5.86E-03	OV
TMEM232	1.36	5.90E-03	OV
HPGDS	-1.27	5.93E-03	OV
C17orf87	-1.07	5.93E-03	OV
KLK10	-1.52	5.93E-03	OV
SELM	-1.28	5.93E-03	OV
SPP1	-1.05	5.93E-03	OV
CD53	-1.21	6.03E-03	OV
CD7	-1.62	6.07E-03	OV
MRC1	-1.38	6.19E-03	OV
BIN2	-1.24	6.24E-03	OV
CD247	-1.40	6.32E-03	OV
PTPRCAP	-1.22	6.38E-03	OV
EVI2B	-1.32	6.38E-03	OV
C3AR1	-1.29	6.41E-03	OV
CLIC5	-1.21	6.41E-03	OV
DOK2	-1.07	6.49E-03	OV
KIAA0748	-1.44	6.49E-03	OV
SASH3	-1.26	6.49E-03	OV
RIPK3	-1.05	6.54E-03	OV
TREM2	-1.33	6.88E-03	OV
CASC1	1.20	7.12E-03	OV
NCKAP1L	-1.28	7.19E-03	OV
ZNF727	1.35	7.19E-03	OV
GPR77	-1.09	7.24E-03	OV
SLA	-1.14	7.24E-03	OV

NCF4	-1.04	7.28E-03	OV
VAV1	-1.16	7.33E-03	OV
DLEC1	1.22	7.52E-03	OV
DPYD	-1.33	7.52E-03	OV
TNFAIP8L2	-1.10	7.52E-03	OV
TNFRSF11A	-1.15	7.52E-03	OV
TLR8	-1.50	7.62E-03	OV
SALL1	1.68	7.65E-03	OV
SLC9A9	-1.04	7.65E-03	OV
IL10RA	-1.15	7.92E-03	OV
PLEK	-1.18	7.96E-03	OV
NKX3-1	-1.34	8.05E-03	OV
FCGR1B	-1.16	8.13E-03	OV
WBSCR26	-2.24	8.15E-03	OV
C10orf128	-1.23	8.27E-03	OV
FCRLB	-1.32	8.27E-03	OV
FGL2	-1.27	8.27E-03	OV
TMEM200A	-1.18	8.31E-03	OV
DARC	-1.60	8.35E-03	OV
FLRT1	1.22	8.37E-03	OV
HK3	-1.24	8.52E-03	OV
SNORA12	-1.39	8.52E-03	OV
NFAM1	-1.08	8.57E-03	OV
MGP	-1.03	8.65E-03	OV
FPR1	-1.40	8.81E-03	OV
PRSS35	-1.59	8.87E-03	OV
APOC1	-1.08	8.88E-03	OV
FCGR2C	-1.48	8.92E-03	OV
HCK	-1.13	8.92E-03	OV
SERPINA1	-1.25	8.92E-03	OV
CLDN11	-1.27	8.94E-03	OV
SAMSN1	-1.16	8.96E-03	OV
RTN1	-1.22	9.10E-03	OV
TFPI2	-1.90	9.10E-03	OV
APBB1IP	-1.29	9.19E-03	OV
COL9A3	1.48	9.30E-03	OV
PTPN7	-1.08	9.42E-03	OV
PRSS33	2.14	9.46E-03	OV
TMEM176B	-1.21	9.46E-03	OV
TMEM150B	-1.32	9.51E-03	OV
C16orf54	-1.23	9.52E-03	OV

LOC441666	1.07	9.60E-03	OV
NCF2	-1.04	9.68E-03	OV
PTCH2	1.01	9.72E-03	OV
LRRIQ1	1.17	9.77E-03	OV
C13orf33	-1.55	1.02E-02	OV
C7orf58	-1.05	1.02E-02	OV
SDS	-1.09	1.02E-02	OV
EPSTI1	-1.11	1.03E-02	OV
IGF1	-1.27	1.03E-02	OV
RERG	-1.53	1.04E-02	OV
DKK2	-1.25	1.04E-02	OV
DNAH2	1.29	1.04E-02	OV
LOC221442	1.10	1.04E-02	OV
IL12RB1	-1.42	1.04E-02	OV
EVI2A	-1.28	1.07E-02	OV
TLR7	-1.27	1.07E-02	OV
MYO1F	-1.05	1.08E-02	OV
MATK	-1.49	1.08E-02	OV
CLEC7A	-1.27	1.12E-02	OV
AOAH	-1.46	1.12E-02	OV
IL32	-1.12	1.13E-02	OV
P2RY8	-1.04	1.13E-02	OV
CD180	-1.10	1.18E-02	OV
CYTIP	-1.05	1.19E-02	OV
LGR5	1.64	1.19E-02	OV
ITGAM	-1.28	1.19E-02	OV
SAMD3	-1.30	1.19E-02	OV
FCRL6	-1.67	1.20E-02	OV
WDFY4	-1.34	1.21E-02	OV
SLC38A5	-1.65	1.22E-02	OV
AMH	-2.00	1.23E-02	OV
MARCO	-2.01	1.23E-02	OV
CD6	-1.17	1.24E-02	OV
MPEG1	-1.07	1.24E-02	OV
FCGR1A	-1.20	1.25E-02	OV
GPR65	-1.09	1.25E-02	OV
HGFAC	1.23	1.25E-02	OV
TNFSF13B	-1.18	1.26E-02	OV
LOC349196	-1.05	1.26E-02	OV
RASAL3	-1.10	1.26E-02	OV
RASL11A	-1.28	1.26E-02	OV

GRAP2	-1.43	1.29E-02	OV
CD33	-1.03	1.30E-02	OV
C14orf49	-1.12	1.30E-02	OV
EMILIN3	1.15	1.31E-02	OV
APOL3	-1.18	1.32E-02	OV
PARP15	-1.20	1.32E-02	OV
SGSM1	1.24	1.33E-02	OV
CD209	-1.17	1.34E-02	OV
PTAFR	-1.03	1.36E-02	OV
CLEC2B	-1.02	1.36E-02	OV
MAN1A1	-1.13	1.41E-02	OV
FCGR1C	-1.13	1.41E-02	OV
C12orf68	-1.02	1.42E-02	OV
RGS18	-1.12	1.43E-02	OV
CD3G	-1.47	1.44E-02	OV
CD84	-1.25	1.44E-02	OV
LILRB4	-1.24	1.44E-02	OV
SERPINF1	-1.24	1.46E-02	OV
TTC9	-1.15	1.46E-02	OV
LOC285830	-1.02	1.47E-02	OV
GAPT	-1.30	1.47E-02	OV
CYSLTR1	-1.18	1.52E-02	OV
GDF1	1.07	1.52E-02	OV
GFAP	1.45	1.52E-02	OV
SIT1	-1.39	1.53E-02	OV
IL2RA	-1.25	1.53E-02	OV
PDGFRA	-1.44	1.54E-02	OV
PTPN22	-1.24	1.54E-02	OV
DOCK2	-1.24	1.60E-02	OV
TAGAP	-1.03	1.61E-02	OV
KCNMB2	1.11	1.62E-02	OV
WNK2	1.01	1.63E-02	OV
ALDH1A1	-1.04	1.64E-02	OV
CD48	-1.28	1.64E-02	OV
HLA-DRA	-1.01	1.64E-02	OV
CLEC10A	-1.68	1.69E-02	OV
SLC22A3	-1.46	1.69E-02	OV
GNLY	-1.98	1.72E-02	OV
PROM1	1.56	1.73E-02	OV
LRRK2	-1.10	1.73E-02	OV
KLHL4	-1.27	1.75E-02	OV

SLAMF8	-1.24	1.75E-02	OV
SSPO	1.12	1.76E-02	OV
ANKRD22	-1.30	1.78E-02	OV
KRT15	-1.67	1.78E-02	OV
COPZ2	-1.15	1.78E-02	OV
SLC24A3	-1.30	1.78E-02	OV
FOLR3	-2.21	1.79E-02	OV
NTN4	-1.03	1.79E-02	OV
C12orf59	-1.17	1.80E-02	OV
LILRB5	-1.08	1.81E-02	OV
GPR82	-1.30	1.84E-02	OV
HSD11B1	-1.23	1.85E-02	OV
TSIX	1.14	1.85E-02	OV
C2orf40	1.20	1.90E-02	OV
ARHGAP15	-1.19	1.95E-02	OV
CCR2	-1.54	1.97E-02	OV
MEI1	-1.33	1.97E-02	OV
FPR3	-1.18	1.99E-02	OV
CFD	-1.07	2.01E-02	OV
FCGR2B	-1.41	2.02E-02	OV
PTPRC	-1.19	2.02E-02	OV
SLAMF6	-1.40	2.03E-02	OV
RARRES3	-1.11	2.03E-02	OV
VCAM1	-1.41	2.07E-02	OV
CHGA	1.60	2.10E-02	OV
LEFTY2	1.44	2.12E-02	OV
CAMK2B	1.09	2.14E-02	OV
FAM81B	1.60	2.14E-02	OV
SNAI2	-1.19	2.14E-02	OV
VTCN1	-1.58	2.14E-02	OV
ARMC3	1.16	2.15E-02	OV
NCF1C	-1.17	2.18E-02	OV
IKZF1	-1.01	2.20E-02	OV
TMEM215	-1.65	2.20E-02	OV
LCN2	-1.38	2.22E-02	OV
CXCR3	-1.37	2.22E-02	OV
FYB	-1.16	2.23E-02	OV
GPR120	-1.24	2.25E-02	OV
KYNU	-1.03	2.25E-02	OV
LILRB2	-1.07	2.26E-02	OV
SLC2A5	-1.05	2.28E-02	OV

RASGRF2	-1.01	2.30E-02	OV
GPR84	-1.20	2.30E-02	OV
ARHGAP20	-1.19	2.33E-02	OV
GZMK	-1.85	2.33E-02	OV
AMICA1	-1.20	2.34E-02	OV
SNX20	-1.02	2.34E-02	OV
CCR5	-1.20	2.34E-02	OV
FMO2	-2.27	2.39E-02	OV
RUNX1T1	-1.44	2.41E-02	OV
S100A3	-1.19	2.42E-02	OV
DDX11L2	-1.42	2.47E-02	OV
PDCD1LG2	-1.18	2.47E-02	OV
CPM	-1.06	2.48E-02	OV
SORCS2	-1.83	2.49E-02	OV
B3GNT3	-1.32	2.51E-02	OV
LRRC16B	1.11	2.59E-02	OV
CBFA2T3	-1.07	2.59E-02	OV
CD5	-1.21	2.60E-02	OV
LOXL4	-1.20	2.72E-02	OV
TLR10	-1.18	2.72E-02	OV
TMEM71	-1.01	2.72E-02	OV
KLK1	-1.80	2.73E-02	OV
WISP1	-1.16	2.75E-02	OV
IL10	-1.04	2.75E-02	OV
SELL	-1.09	2.75E-02	OV
PDLIM3	-1.17	2.79E-02	OV
KLRK1	-1.50	2.81E-02	OV
SIGLEC10	-1.16	2.83E-02	OV
SECTM1	-1.17	2.84E-02	OV
KLK13	-1.45	2.86E-02	OV
FOXQ1	-1.44	2.88E-02	OV
GZMA	-1.55	2.89E-02	OV
CA8	1.22	2.90E-02	OV
ADAM33	1.06	2.92E-02	OV
DPEP1	-1.68	2.94E-02	OV
BCAT1	1.31	2.95E-02	OV
PLA2G2A	-2.29	3.02E-02	OV
HGF	-1.03	3.08E-02	OV
LTA	-1.06	3.10E-02	OV
TFAP2C	-1.12	3.10E-02	OV
MNDA	-1.03	3.13E-02	OV

DEFB1	-2.38	3.14E-02	OV
IGSF21	-1.20	3.14E-02	OV
CSF2RB	-1.06	3.14E-02	OV
DNAI1	1.34	3.16E-02	OV
SLA2	-1.08	3.17E-02	OV
RGS6	-1.15	3.21E-02	OV
CCL3	-1.02	3.23E-02	OV
PRPH	1.10	3.25E-02	OV
OSR1	-1.30	3.26E-02	OV
DIRAS1	-1.49	3.27E-02	OV
TSPAN7	1.05	3.32E-02	OV
CLEC12A	-1.32	3.38E-02	OV
ATP1A3	-2.06	3.40E-02	OV
ANXA8L2	-1.67	3.41E-02	OV
AIM2	-1.38	3.43E-02	OV
SLC22A18AS	-1.11	3.43E-02	OV
SFRP4	-1.67	3.44E-02	OV
CHRNB4	-1.19	3.47E-02	OV
MGAT5B	1.59	3.50E-02	OV
ADAMTS8	1.01	3.50E-02	OV
RHD	1.13	3.53E-02	OV
FGFR3	1.05	3.57E-02	OV
PDZK1IP1	-1.52	3.61E-02	OV
RGS4	-1.10	3.64E-02	OV
VWA3A	1.45	3.73E-02	OV
CCL5	-1.31	3.77E-02	OV
PRF1	-1.11	3.77E-02	OV
ATP1B2	1.07	3.79E-02	OV
CLDN10	-2.19	3.82E-02	OV
CALML5	-2.30	3.84E-02	OV
BTC	-1.12	3.85E-02	OV
CRTAM	-1.21	3.86E-02	OV
CD96	-1.03	3.87E-02	OV
TIMP4	-1.39	3.89E-02	OV
HLA-DQA1	-1.33	3.93E-02	OV
XKR9	-1.31	3.94E-02	OV
EFCAB4B	-1.02	3.95E-02	OV
UBASH3A	-1.23	3.97E-02	OV
IL2RG	-1.19	3.98E-02	OV
STEAP4	-1.34	4.01E-02	OV
LUM	-1.30	4.02E-02	OV

TNIK	-1.31	4.03E-02	OV
CEACAM6	-2.08	4.06E-02	OV
PTPRN	-1.21	4.06E-02	OV
IBSP	-1.52	4.08E-02	OV
ITK	-1.37	4.12E-02	OV
CD2	-1.53	4.18E-02	OV
SLC10A4	1.02	4.18E-02	OV
LYVE1	-1.04	4.18E-02	OV
MSC	-1.27	4.18E-02	OV
IL21R	-1.69	4.22E-02	OV
FASLG	-1.25	4.24E-02	OV
XCL1	-1.13	4.27E-02	OV
NTRK2	-1.12	4.29E-02	OV
TCF21	-1.63	4.40E-02	OV
CLEC5A	-1.07	4.47E-02	OV
OSM	-1.05	4.47E-02	OV
FBN3	1.40	4.48E-02	OV
MEIS3	-1.12	4.50E-02	OV
IL2RB	-1.24	4.63E-02	OV
KRT4	-2.18	4.64E-02	OV
KLK7	-1.00	4.64E-02	OV
CD8B	-1.31	4.65E-02	OV
GPR1	-1.19	4.65E-02	OV
ITGB6	-1.43	4.70E-02	OV
CLEC4E	-1.34	4.71E-02	OV
THSD7A	1.01	4.73E-02	OV
PREX2	1.01	4.75E-02	OV
CCL14	-1.34	4.84E-02	OV
EBI3	-1.01	4.90E-02	OV
IMPG2	1.52	4.98E-02	OV
A2ML1	1.29	1.47E-05	BRCA
ABAT	-1.08	1.47E-10	BRCA
ABCA12	-1.61	2.98E-07	BRCA
ABCA6	-1.23	3.81E-11	BRCA
ABCA8	-1.24	2.07E-05	BRCA
ABCA9	-1.29	3.85E-09	BRCA
ABCC11	-1.33	2.76E-04	BRCA
ABCC6	-1.10	8.29E-09	BRCA
ABCC8	-1.41	2.47E-05	BRCA
ABCC9	-1.02	1.52E-13	BRCA
ABCG2	-1.06	1.20E-14	BRCA

ACCN3	1.01	5.56E-14	BRCA
ACER2	-1.27	3.38E-20	BRCA
ACOX2	-1.10	8.74E-08	BRCA
ACVR1C	-1.02	5.32E-06	BRCA
ADAM22	-1.26	2.22E-15	BRCA
ADAMTS15	-1.68	1.17E-11	BRCA
ADAMTS18	-1.06	9.76E-07	BRCA
ADAT3	1.02	1.69E-25	BRCA
ADCK5	1.02	1.88E-36	BRCA
ADCY5	-1.35	2.28E-07	BRCA
ADH1B	-1.86	2.19E-04	BRCA
ADIPOQ	-2.13	3.07E-05	BRCA
AFF3	-1.38	2.49E-09	BRCA
AGER	1.04	3.22E-28	BRCA
AGR2	-1.48	4.41E-06	BRCA
AGR3	-2.01	3.90E-05	BRCA
AGTR1	-1.47	2.33E-07	BRCA
AIM1L	1.04	4.00E-09	BRCA
AK5	-1.32	2.14E-07	BRCA
AKAP12	-1.02	2.26E-12	BRCA
AKAP2	-1.08	6.67E-12	BRCA
ALOX12P2	1.11	1.07E-06	BRCA
AMPH	-1.07	3.73E-10	BRCA
ANGPTL1	-1.20	1.46E-10	BRCA
ANGPTL6	1.05	9.30E-17	BRCA
ANKRD30A	-2.91	2.97E-08	BRCA
ANKRD30B	-1.24	1.28E-04	BRCA
ANKRD43	-1.22	4.53E-07	BRCA
ANO3	-1.33	4.49E-07	BRCA
ANO5	-1.18	2.77E-07	BRCA
AQP7	-1.13	2.52E-04	BRCA
AQPEP	-1.00	4.22E-06	BRCA
ARHGAP20	-1.18	5.08E-14	BRCA
ARHGEF38	-1.20	2.55E-10	BRCA
AR	-2.15	6.63E-14	BRCA
ASPA	-1.11	1.50E-09	BRCA
ATAD3B	1.04	6.56E-35	BRCA
ATP1A2	-1.15	2.65E-05	BRCA
ATP1A4	-1.89	2.59E-15	BRCA
AURKB	1.41	6.67E-26	BRCA
AVPR1A	-1.28	3.08E-17	BRCA

B3GALT5	-1.14	3.68E-06	BRCA
B3GNT4	1.08	2.22E-13	BRCA
BAIAP2L2	1.33	2.08E-20	BRCA
BARX1	1.28	8.79E-10	BRCA
BIRC5	1.26	3.10E-18	BRCA
C14orf73	1.26	1.35E-15	BRCA
C16orf59	1.02	8.34E-21	BRCA
C16orf79	1.21	1.75E-25	BRCA
C18orf56	1.14	3.59E-22	BRCA
C19orf20	1.25	2.83E-23	BRCA
C19orf60	1.09	7.41E-40	BRCA
C1orf106	1.01	1.73E-07	BRCA
C20orf151	1.01	8.04E-19	BRCA
C21orf70	1.05	1.21E-39	BRCA
C2CD4D	1.13	8.16E-26	BRCA
C2orf48	1.13	5.48E-12	BRCA
C4orf48	1.43	1.87E-24	BRCA
C6orf108	1.10	7.41E-40	BRCA
C6orf223	1.10	4.59E-07	BRCA
C8ORFK29	1.07	1.83E-15	BRCA
CALML5	1.52	8.00E-08	BRCA
CAPS	1.21	7.38E-18	BRCA
CBS	1.15	7.45E-14	BRCA
CBX2	1.01	8.55E-13	BRCA
CCDC154	1.09	3.02E-15	BRCA
CCDC42B	1.09	8.63E-14	BRCA
CCDC78	1.03	8.24E-10	BRCA
CCDC85B	1.04	1.37E-26	BRCA
CCDC88B	1.00	2.08E-25	BRCA
CCL3L1	1.19	2.00E-15	BRCA
CCNE1	1.12	1.19E-17	BRCA
CD19	1.17	6.51E-07	BRCA
CDC20	1.17	1.45E-17	BRCA
CDC45	1.12	2.00E-16	BRCA
CDCA3	1.02	1.91E-18	BRCA
CDKN2A	1.37	1.07E-23	BRCA
CDT1	1.24	5.97E-28	BRCA
CENPA	1.05	3.05E-15	BRCA
CENPM	1.09	1.53E-21	BRCA
CHODL	1.13	2.07E-08	BRCA
CHTF18	1.07	5.39E-31	BRCA

CLDN3	1.02	2.25E-11	BRCA
CLEC18A	1.14	3.56E-20	BRCA
CLEC18B	1.10	2.13E-14	BRCA
CLIC3	1.07	7.13E-13	BRCA
COL11A2	1.43	5.77E-17	BRCA
COL2A1	1.14	3.77E-04	BRCA
COL9A3	1.55	1.04E-15	BRCA
COMTD1	1.17	1.79E-32	BRCA
CRABP1	1.12	2.60E-04	BRCA
CRLF1	1.01	1.08E-09	BRCA
CYP2D7P1	1.03	8.60E-19	BRCA
DDN	1.09	1.44E-12	BRCA
DGCR5	1.09	1.02E-12	BRCA
DNER	1.04	1.15E-05	BRCA
DUSP9	1.04	2.36E-06	BRCA
E2F1	1.02	1.46E-20	BRCA
EMID2	1.09	1.98E-10	BRCA
EN1	1.32	1.35E-12	BRCA
ENHO	1.24	6.65E-15	BRCA
ESPNL	1.10	2.15E-12	BRCA
FAM131C	1.35	1.01E-15	BRCA
FAM64A	1.06	4.54E-16	BRCA
FBN3	1.09	4.29E-06	BRCA
FER1L4	1.04	2.60E-13	BRCA
FOXD1	1.20	7.10E-10	BRCA
FOXH1	1.25	2.20E-20	BRCA
FUT7	1.15	7.21E-18	BRCA
FZD9	1.21	2.50E-10	BRCA
GAL	1.40	5.56E-11	BRCA
GFRA3	1.05	4.28E-07	BRCA
GJB3	1.56	4.27E-14	BRCA
GJB5	1.62	2.52E-16	BRCA
GLDC	1.21	2.37E-07	BRCA
GLI4	1.11	6.11E-37	BRCA
GPC2	1.30	3.06E-20	BRCA
GSDMC	1.16	5.75E-10	BRCA
GSTM1	2.26	1.57E-11	BRCA
HAGHL	1.21	9.39E-17	BRCA
HES4	1.41	2.44E-34	BRCA
HIST1H2AM	1.04	6.28E-11	BRCA
HIST1H2BO	1.09	2.65E-11	BRCA

HIST2H3C	1.02	8.28E-12	BRCA
HIST3H2A	1.08	6.66E-11	BRCA
HOMER3	1.04	5.30E-37	BRCA
HPDL	1.21	7.67E-14	BRCA
IER5L	1.10	1.83E-29	BRCA
ISG15	1.29	2.42E-16	BRCA
JSRP1	1.85	2.68E-22	BRCA
KCNG1	1.48	7.19E-12	BRCA
KIF18B	1.16	5.81E-17	BRCA
KIF1A	1.04	7.01E-04	BRCA
KIFC2	1.03	7.96E-25	BRCA
KRT16	1.70	6.55E-11	BRCA
KRT6A	1.21	7.26E-05	BRCA
KRT6B	1.08	4.39E-04	BRCA
KRT6C	1.41	6.44E-08	BRCA
KRT81	1.61	1.11E-10	BRCA
KRT86	1.10	1.08E-09	BRCA
LCN2	1.01	7.38E-05	BRCA
LGALS4	1.22	2.19E-17	BRCA
LGALS7	1.02	5.97E-06	BRCA
LIME1	1.26	1.31E-24	BRCA
LOC339535	1.15	5.33E-07	BRCA
LOC400696	1.36	4.96E-12	BRCA
LTB	1.21	1.15E-13	BRCA
LYG1	1.03	6.15E-21	BRCA
MEF2B	1.28	1.13E-28	BRCA
MIA	1.14	1.33E-05	BRCA
MIF	1.08	7.65E-25	BRCA
MND1	1.04	5.09E-14	BRCA
MSLN	2.43	1.82E-18	BRCA
MST1P9	1.15	9.17E-15	BRCA
MYBL2	1.28	1.51E-15	BRCA
MYLK2	1.32	1.59E-20	BRCA
NACA2	1.08	8.95E-35	BRCA
NCRNA00105	1.02	3.41E-20	BRCA
NFKBIL2	1.01	1.01E-25	BRCA
NOTUM	1.30	1.79E-12	BRCA
NOXO1	1.14	6.32E-15	BRCA
NPM2	1.05	1.95E-09	BRCA
NRTN	1.34	6.88E-17	BRCA
NSUN5P1	1.11	2.61E-39	BRCA

ORC6L	1.01	2.46E-18	BRCA
P2RY11	1.59	9.52E-15	BRCA
PAQR6	1.05	9.54E-19	BRCA
PCSK1N	2.18	1.12E-19	BRCA
PIF1	1.55	3.09E-39	BRCA
PITX1	1.00	8.57E-07	BRCA
PKMYT1	1.44	1.04E-22	BRCA
POMC	1.07	4.31E-15	BRCA
PPP1R14C	1.07	1.95E-06	BRCA
PRAME	1.46	2.76E-05	BRCA
PRR22	1.00	2.28E-23	BRCA
PRR7	1.30	1.53E-26	BRCA
PRSS50	1.23	6.44E-10	BRCA
RAD54L	1.06	4.87E-18	BRCA
RBP1	1.15	8.73E-16	BRCA
RCOR2	1.09	1.52E-13	BRCA
RECQL4	1.16	1.69E-24	BRCA
RFPL3S	1.14	1.79E-25	BRCA
RLTPR	1.21	1.15E-13	BRCA
RNF112	1.01	9.57E-17	BRCA
ROPN1B	1.18	1.64E-05	BRCA
RUFY4	1.36	2.00E-16	BRCA
SCAND1	1.01	9.69E-33	BRCA
SCT	1.05	4.16E-08	BRCA
SH2B2	1.00	2.55E-22	BRCA
SH3D20	1.10	6.22E-19	BRCA
SPC24	1.16	1.76E-14	BRCA
SPIB	1.08	1.22E-07	BRCA
SYCE1L	1.65	2.48E-18	BRCA
SYT8	1.36	2.51E-07	BRCA
TCAP	1.26	1.16E-16	BRCA
TMEM105	1.05	7.59E-09	BRCA
TMEM191A	1.05	7.85E-26	BRCA
TMPRSS5	1.12	3.04E-13	BRCA
TNFRSF25	1.13	3.82E-27	BRCA
TNFRSF4	1.08	8.77E-26	BRCA
TNFRSF6B	1.01	2.12E-23	BRCA
TNNI2	1.03	7.64E-11	BRCA
TNNT1	1.42	9.17E-08	BRCA
TPRXL	1.07	4.23E-09	BRCA
TREX2	1.05	9.30E-17	BRCA

TROAP	1.20	6.50E-19	BRCA
TSIX	1.68	1.96E-17	BRCA
TSPAN10	1.21	1.46E-22	BRCA
TSPO	1.02	4.28E-38	BRCA
TUBB8	1.21	1.72E-24	BRCA
UBE2C	1.24	2.17E-16	BRCA
UBE2S	1.12	1.52E-30	BRCA
UPK3BL	1.11	5.26E-13	BRCA
UPK3B	1.34	2.04E-18	BRCA
VGF	1.75	6.32E-18	BRCA
VGLL1	1.12	4.87E-04	BRCA
WNT6	1.60	2.15E-15	BRCA
YJEFN3	1.09	9.42E-20	BRCA
ZNHIT2	1.03	6.80E-08	BRCA
BAGE2	-1.07	1.69E-11	BRCA
BCAS1	-1.08	1.10E-04	BRCA
BICC1	-1.40	1.56E-14	BRCA
BMPR1B	-1.10	2.26E-03	BRCA
BPIL1	-1.25	4.10E-04	BRCA
BTBD8	-1.25	1.11E-15	BRCA
C1QTNF7	-1.26	3.91E-11	BRCA
C1orf150	-1.28	5.07E-13	BRCA
C1orf173	-1.12	5.71E-05	BRCA
C1orf64	-1.95	4.13E-06	BRCA
C20orf114	-1.12	2.13E-03	BRCA
C20orf94	-1.02	4.23E-16	BRCA
C4orf31	-1.34	1.64E-12	BRCA
C6orf97	-1.13	8.76E-09	BRCA
C6	-1.11	1.23E-04	BRCA
C7orf58	-1.20	3.80E-13	BRCA
C8orf34	-1.03	1.50E-07	BRCA
CA12	-1.26	2.44E-10	BRCA
CACNA2D1	-2.11	1.08E-19	BRCA
CADM2	-1.08	1.86E-06	BRCA
CAPN8	-1.12	4.29E-04	BRCA
CASQ1	-1.11	1.09E-09	BRCA
CASQ2	-1.02	2.46E-07	BRCA
CCDC144A	-1.20	4.19E-11	BRCA
CCDC158	-1.44	7.79E-13	BRCA
CCDC160	-1.12	1.00E-09	BRCA
CCNT1	-1.36	1.97E-19	BRCA

CD36	-1.24	2.62E-07	BRCA
CDKL5	-1.32	9.50E-14	BRCA
CEACAM5	-1.16	3.20E-03	BRCA
CES1	-1.36	7.63E-08	BRCA
CHAD	-1.20	9.73E-05	BRCA
CHL1	-1.35	5.93E-09	BRCA
CIDEA	-1.30	3.35E-04	BRCA
CIDEC	-1.18	1.32E-03	BRCA
CLGN	-1.06	1.58E-05	BRCA
CLIC6	-1.46	2.77E-06	BRCA
CLSTN2	-1.35	6.03E-09	BRCA
CNTN1	-1.44	5.12E-11	BRCA
CNTN4	-1.14	3.86E-09	BRCA
CNTNAP2	-1.16	3.34E-04	BRCA
COL14A1	-1.13	6.56E-12	BRCA
CPA3	-1.08	2.26E-06	BRCA
CPB1	-1.96	4.58E-04	BRCA
CSRNP3	-1.25	6.35E-12	BRCA
CST1	-1.16	2.63E-03	BRCA
CST2	-1.55	1.20E-08	BRCA
CT62	-1.30	9.98E-08	BRCA
CTTNBP2	-1.15	2.08E-10	BRCA
CX3CR1	-1.08	3.14E-10	BRCA
CYP2A6	-1.36	2.00E-04	BRCA
CYP2B7P1	-1.77	1.02E-04	BRCA
CYP4B1	-1.07	1.54E-04	BRCA
CYP4X1	-1.25	4.86E-06	BRCA
CYP4Z1	-2.06	3.26E-06	BRCA
CYP4Z2P	-1.67	9.24E-07	BRCA
DACH1	-1.28	1.28E-07	BRCA
DCDC2	-1.12	2.74E-05	BRCA
DCLK1	-1.25	1.57E-11	BRCA
DDI2	-1.18	5.59E-17	BRCA
DDR2	-1.11	6.53E-11	BRCA
DEFB132	-1.33	1.28E-06	BRCA
DNAH5	-1.22	8.09E-09	BRCA
DNAH7	-1.13	1.77E-13	BRCA
DOC2B	-1.04	1.24E-07	BRCA
DYNC2H1	-1.18	1.70E-21	BRCA
EBF2	-1.20	2.97E-12	BRCA
EDIL3	-1.49	5.24E-13	BRCA

ENTPD3	-1.07	3.05E-10	BRCA
EPHA3	-1.03	5.57E-10	BRCA
ERBB4	-1.99	3.83E-10	BRCA
ESR1	-1.81	3.12E-12	BRCA
EYA4	-1.10	2.85E-07	BRCA
F13A1	-1.01	1.74E-10	BRCA
F7	-1.22	1.84E-06	BRCA
FABP4	-1.17	2.20E-03	BRCA
FAIM2	-1.05	1.18E-07	BRCA
FAM106C	-1.16	3.64E-13	BRCA
FAM184B	-1.29	4.60E-07	BRCA
FAM196B	-1.10	4.65E-06	BRCA
FAM198B	-1.09	1.05E-19	BRCA
FAM63B	-1.04	3.44E-19	BRCA
FAT3	-1.59	3.02E-20	BRCA
FAT4	-1.09	7.59E-14	BRCA
FGF10	-1.80	2.13E-10	BRCA
FHL5	-1.17	5.80E-13	BRCA
FLRT3	-1.09	6.93E-07	BRCA
FMN1	-1.21	1.47E-14	BRCA
FMO2	-1.02	2.18E-05	BRCA
FOXA1	-1.47	7.00E-09	BRCA
FOXP2	-1.08	8.68E-09	BRCA
GABRB3	-1.67	1.55E-10	BRCA
GALNT5	-1.09	2.31E-08	BRCA
GCNT4	-1.10	1.74E-12	BRCA
GFRA1	-1.96	5.84E-11	BRCA
GLDN	-1.01	4.49E-09	BRCA
GLRB	-1.10	2.99E-11	BRCA
GP2	-2.24	1.76E-07	BRCA
GPD1	-1.06	1.83E-03	BRCA
GPR81	-1.42	3.74E-12	BRCA
GPR98	-1.09	1.25E-04	BRCA
GRIK3	-1.07	7.58E-05	BRCA
GRPR	-1.99	4.99E-10	BRCA
GTF2A1	-1.12	4.42E-20	BRCA
GUSBP3	-1.03	5.86E-15	BRCA
HEPACAM2	-1.47	1.21E-06	BRCA
HIPK3	-1.21	1.73E-20	BRCA
HMCN1	-1.03	1.30E-11	BRCA
HMGCS2	-2.16	4.36E-06	BRCA

HRASLS5	-1.19	7.84E-06	BRCA
HRNR	-1.08	1.71E-11	BRCA
HS6ST3	-1.17	8.11E-05	BRCA
HTR2B	-1.04	1.36E-16	BRCA
IGF1	-1.03	5.11E-08	BRCA
IGSF9B	-1.20	4.49E-09	BRCA
IL20RA	-1.59	3.07E-12	BRCA
IL33	-1.05	1.10E-05	BRCA
IL6ST	-2.07	2.54E-23	BRCA
INPP4B	-1.05	8.24E-11	BRCA
IPMK	-1.04	2.01E-14	BRCA
ITGA8	-1.17	1.04E-10	BRCA
ITPR1	-1.08	1.60E-18	BRCA
KCND3	-1.77	1.87E-14	BRCA
KCNJ3	-1.62	1.20E-04	BRCA
KERA	-1.10	1.26E-08	BRCA
KIAA0754	-1.16	9.30E-12	BRCA
KIAA1244	-1.08	9.50E-13	BRCA
KIAA1377	-1.08	4.13E-13	BRCA
KLHDC7A	-1.47	1.59E-05	BRCA
KLHL11	-1.17	4.68E-19	BRCA
KL	-1.18	2.15E-15	BRCA
LAMA2	-1.10	2.48E-11	BRCA
LCOR	-1.17	2.71E-18	BRCA
LEP	-1.31	5.43E-04	BRCA
LGALS12	-1.21	2.70E-05	BRCA
LNPEP	-1.12	6.26E-20	BRCA
LOC100272216	-1.07	2.30E-15	BRCA
LOC145837	-1.07	7.66E-06	BRCA
LOC148696	-1.34	1.27E-14	BRCA
LOC728606	-1.14	1.20E-04	BRCA
LONRF2	-1.31	5.89E-09	BRCA
LPHN3	-1.09	2.66E-08	BRCA
LRP1B	-1.25	3.54E-05	BRCA
LRRC31	-2.30	1.10E-10	BRCA
LRRC37A2	-1.13	3.39E-34	BRCA
LUZP2	-1.09	1.17E-06	BRCA
MAN2A1	-1.04	1.82E-19	BRCA
MAOA	-1.10	1.54E-06	BRCA
MAST4	-1.09	1.10E-14	BRCA
MBNL3	-1.02	3.68E-11	BRCA

MGAT5	-1.07	5.47E-13	BRCA
MICALCL	-1.16	1.15E-13	BRCA
MKX	-1.09	5.65E-06	BRCA
MMRN1	-1.18	6.35E-07	BRCA
MS4A2	-1.21	1.55E-09	BRCA
MUC6	-1.01	9.81E-03	BRCA
MYH11	-1.07	4.21E-07	BRCA
MYO9A	-1.03	1.88E-19	BRCA
MYOCD	-1.26	3.66E-12	BRCA
MYRIP	-1.46	3.03E-13	BRCA
N4BP2	-1.04	1.10E-15	BRCA
NAT1	-1.44	8.08E-08	BRCA
NAV3	-1.04	1.42E-09	BRCA
NBEAL1	-1.24	2.49E-17	BRCA
NBEA	-1.14	9.14E-15	BRCA
NCAM2	-1.42	4.21E-10	BRCA
NCOA2	-1.02	2.86E-13	BRCA
NEK10	-1.44	8.16E-07	BRCA
NEK5	-1.03	4.62E-08	BRCA
NELL2	-1.09	1.29E-05	BRCA
NFATC2	-1.17	3.10E-12	BRCA
NHSL2	-1.35	7.51E-22	BRCA
NOVA1	-1.29	3.59E-07	BRCA
NRXN3	-1.03	9.37E-10	BRCA
NTN4	-1.17	4.64E-12	BRCA
NTRK2	-1.15	2.30E-07	BRCA
ODZ2	-1.05	1.97E-06	BRCA
OGN	-1.12	3.97E-05	BRCA
OMD	-1.07	7.09E-08	BRCA
P2RY12	-1.01	1.53E-08	BRCA
P2RY1	-1.00	4.00E-16	BRCA
PAR5	-1.42	1.17E-14	BRCA
PARD3B	-1.22	1.06E-18	BRCA
PCDHA4	-1.01	1.22E-05	BRCA
PCDHA6	-1.17	2.73E-07	BRCA
PCDHGA2	-1.07	8.77E-10	BRCA
PCDHGA4	-1.02	6.36E-10	BRCA
PCDHGA9	-1.00	1.63E-11	BRCA
PCDHGB7	-1.18	1.31E-13	BRCA
PCK1	-1.42	4.08E-05	BRCA
PDE11A	-1.15	1.54E-10	BRCA

PDE3A	-1.22	1.71E-13	BRCA
PDE8B	-1.07	1.44E-08	BRCA
PDK4	-1.15	9.16E-09	BRCA
PGR	-1.81	2.87E-07	BRCA
PIP	-1.68	9.07E-04	BRCA
PIWIL2	-1.07	1.24E-09	BRCA
PLCXD3	-1.10	4.41E-06	BRCA
PLIN1	-1.26	3.93E-04	BRCA
PLXNC1	-1.19	4.38E-13	BRCA
PPAN-P2RY11	-1.56	1.32E-05	BRCA
PPFIA2	-1.01	3.94E-11	BRCA
PREX2	-1.84	6.25E-19	BRCA
PRG4	-1.01	7.84E-05	BRCA
PRKG1	-1.40	2.14E-17	BRCA
PRKG2	-1.42	6.08E-16	BRCA
PRND	-1.04	2.15E-09	BRCA
PRSS21	-1.65	9.28E-09	BRCA
PTPRT	-1.76	7.92E-07	BRCA
RAB27B	-1.29	5.36E-15	BRCA
RAB30	-1.14	4.47E-15	BRCA
RALGAPA2	-1.31	1.00E-19	BRCA
RANBP3L	-1.13	9.79E-07	BRCA
RAPGEF6	-1.10	2.72E-17	BRCA
RASEF	-1.38	2.75E-11	BRCA
RASSF6	-1.05	1.01E-07	BRCA
RBMS3	-1.03	4.09E-13	BRCA
RERGL	-1.08	7.62E-05	BRCA
REST	-1.02	4.12E-16	BRCA
RGPD5	-1.12	7.83E-15	BRCA
RGS22	-1.56	1.18E-08	BRCA
RGS7BP	-1.25	4.61E-11	BRCA
ROBO2	-1.51	9.57E-08	BRCA
RORB	-1.06	2.93E-09	BRCA
RPS28	-1.49	5.49E-06	BRCA
RPS6KA6	-1.05	6.03E-08	BRCA
RUNDC2A	-1.03	5.87E-20	BRCA
RUNX1T1	-1.22	1.70E-14	BRCA
SCGB1D2	-1.62	1.29E-03	BRCA
SCGB2A2	-2.15	1.80E-04	BRCA
SCN2B	-1.28	2.61E-08	BRCA
SCN3A	-1.11	5.59E-08	BRCA

SCN7A	-2.12	5.02E-17	BRCA
SCUBE2	-1.74	1.04E-11	BRCA
SEMA3D	-1.04	1.13E-07	BRCA
SEMA3E	-1.42	1.86E-08	BRCA
SEMA5A	-1.07	7.95E-17	BRCA
SERINC5	-1.23	1.30E-15	BRCA
SERPINA11	-1.70	2.23E-06	BRCA
SLC13A2	-1.01	8.22E-04	BRCA
SLC14A2	-1.38	1.86E-11	BRCA
SLC16A12	-1.28	4.81E-10	BRCA
SLC16A6	-1.08	9.01E-07	BRCA
SLC16A7	-1.26	1.57E-13	BRCA
SLC18A2	-1.23	4.11E-10	BRCA
SLC24A2	-1.00	3.23E-05	BRCA
SLC30A8	-1.28	1.21E-03	BRCA
SLC38A11	-1.16	2.48E-12	BRCA
SLC40A1	-1.29	8.09E-16	BRCA
SLC44A4	-1.37	1.18E-06	BRCA
SLC44A5	-1.09	2.36E-06	BRCA
SLC4A4	-1.05	8.98E-07	BRCA
SLC7A2	-1.62	7.22E-11	BRCA
SLIT2	-1.29	6.73E-14	BRCA
SLITRK6	-1.43	2.18E-05	BRCA
SNORD116-4	-1.38	1.59E-08	BRCA
SORCS1	-1.48	2.94E-06	BRCA
SOX5	-1.09	4.91E-10	BRCA
SPATA18	-1.12	1.88E-12	BRCA
ST8SIA6	-1.82	2.40E-14	BRCA
STK32B	-1.11	4.96E-08	BRCA
SVEP1	-1.19	2.19E-10	BRCA
SYNPO2	-1.00	4.37E-11	BRCA
SYT1	-1.16	1.83E-05	BRCA
SYT9	-1.74	1.19E-07	BRCA
SYTL5	-1.26	1.46E-05	BRCA
TAOK1	-1.22	1.32E-15	BRCA
TAT	-1.07	8.33E-03	BRCA
TBC1D9	-1.07	7.91E-13	BRCA
TDRD1	-1.11	3.31E-05	BRCA
TFAP2B	-1.65	3.32E-04	BRCA
TFF1	-2.18	8.78E-05	BRCA
TFF3	-1.28	2.23E-04	BRCA

TGFBR3	-1.10	8.06E-10	BRCA
THRSP	-1.22	3.26E-05	BRCA
THSD4	-1.02	5.41E-09	BRCA
THSD7A	-1.04	1.43E-13	BRCA
THSD7B	-1.25	7.84E-11	BRCA
TIMP4	-1.18	1.44E-05	BRCA
TLL1	-1.17	1.73E-13	BRCA
TMC5	-1.78	5.29E-09	BRCA
TMEM132C	-1.01	1.40E-03	BRCA
TMTC1	-1.12	1.20E-09	BRCA
TNIK	-1.05	3.26E-11	BRCA
TNNI3K	-1.05	7.90E-10	BRCA
TOX3	-1.21	8.13E-06	BRCA
TP63	-1.01	9.09E-05	BRCA
TPRG1	-1.19	9.57E-06	BRCA
TRIM58	-1.02	3.72E-06	BRCA
TSHZ2	-1.23	1.68E-12	BRCA
TTBK2	-1.14	1.05E-22	BRCA
TUSC5	-1.68	3.39E-05	BRCA
UGCG	-1.12	1.49E-13	BRCA
UHMK1	-1.20	1.53E-14	BRCA
UNC5C	-1.10	6.91E-12	BRCA
VWA2	-1.23	2.57E-07	BRCA
WDR65	-1.29	1.11E-11	BRCA
ZBTB16	-1.38	4.43E-07	BRCA
ZFHX4	-1.05	8.77E-14	BRCA
ZNF192	-1.13	6.82E-16	BRCA
ZNF214	-1.14	4.85E-14	BRCA
ZNF354C	-1.34	1.28E-18	BRCA
ZNF366	-1.15	1.86E-15	BRCA
ZNF676	-1.24	7.01E-09	BRCA
ZNF699	-1.16	7.90E-20	BRCA
ZNF727	-1.07	6.75E-08	BRCA

Gene: gene name

LogFC AA vs EA; Log2 (mean EA tumor RPKM) / (mean AA tumor RPKM) for each transcript

adj.P.Val: P value adjusted for multiple hypothesis testing with BH method

Tumor: CESC = cervix, UCEC = uterine endometrial carcinoma, OV = ovary, BRCA = breast
