# Supplementary material for: Race-associated Molecular Changes in Gynecologic Malignancies
Source: Cancer Res Commun. 2022 Feb 17;2(2):99–109. doi: 10.1158/2767-9764.CRC-21-0018 (PMC9390975; doi:10.1158/2767-9764.CRC-21-0018)
Supplement: Supplemental Table 3 — Gene Set Enrichment Analysis of transcriptional changes in AA samples [file crc-21-0018-s03.pdf]

Supplemental Table 3

Name	Size	NES	FDR q-val	AA vs EA
HALLMARK E2F TARGETS	193	2.81	<0.010	Upregulated
KEGG RIBOSOME	83	2.74	<0.010	Upregulated
GO RRNA METABOLIC PROCESS	239	2.73	<0.010	Upregulated
REACTOME PEPTIDE CHAIN ELONGATION	82	2.72	<0.010	Upregulated
GO ANAPHASE PROMOTING COMPLEX DEPENDENT CATABOLIC PROCESS	75	2.70	<0.010	Upregulated
REACTOME TRANSLATION	143	2.70	<0.010	Upregulated
REACTOME DNA REPLICATION	179	2.69	<0.010	Upregulated
REACTOME NONSENSE MEDIATED DECAY ENHANCED BY THE EXON JUNCTION COMPLEX	101	2.69	<0.010	Upregulated

REACTOME SRP DEPENDENT COTRANSLATIONAL PROTEIN TARGETING TO MEMBRANE	106	2.69	<0.010	Upregulated
REACTOME INFLUENZA VIRAL RNA TRANSCRIPTION AND REPLICATION	98	2.68	<0.010	Upregulated
GO NCRNA PROCESSING	365	2.63	<0.010	Upregulated
REACTOME CELL CYCLE	355	2.62	<0.010	Upregulated
REACTOME 3 UTR MEDIATED TRANSLATIONAL REGULATION	102	2.58	<0.010	Upregulated
GO RIBONUCLEOPROTEIN COMPLEX BIOGENESIS	408	2.58	<0.010	Upregulated
GO OXIDATIVE PHOSPHORYLATION	82	2.57	<0.010	Upregulated

HALLMARK MYC TARGETS V1	195	2.56	<0.010	Upregulated
REACTOME SYNTHESIS OF DNA	83	2.55	<0.010	Upregulated
GO TRANSLATIONAL ELONGATION	110	2.53	<0.010	Upregulated
GO HOMOPHILIC CELL ADHESION VIA PLASMA MEMBRANE ADHESION MOLECULES	108	-2.71	<0.010	Downregulated
HALLMARK UV RESPONSE DN	143	-2.61	<0.010	Downregulated
ATATGCA MIR448	191	-2.46	<0.010	Downregulated
TGCTTTG MIR330	279	-2.42	<0.010	Downregulated
CTTGTAT MIR381	192	-2.36	<0.010	Downregulated
GO CARDIAC CONDUCTION	63	-2.36	<0.010	Downregulated
TAATAAT MIR126	188	-2.35	<0.010	Downregulated

GO REGULATION OF BLOOD CIRCULATION	202	-2.33	<0.010	Downregulated
TGAATGT MIR181A MIR181B MIR181C MIR181D	433	-2.33	<0.010	Downregulated
GO REGULATION OF HEART CONTRACTION	153	-2.33	<0.010	Downregulated
GO MULTICELLULAR ORGANISMAL SIGNALING	88	-2.31	<0.010	Downregulated
CATTTCA MIR203	259	-2.28	<0.010	Downregulated
TTGCACT MIR130A MIR301 MIR130B	369	-2.28	<0.010	Downregulated
GO REGULATION OF PHOSPHATIDYLINOSITOL 3 KINASE SIGNALING	110	-2.28	<0.010	Downregulated

GO MEMBRANE DEPOLARIZATION DURING ACTION POTENTIAL	26	-2.27	<0.010	Downregulated
TAGCTTT MIR9	211	-2.27	<0.010	Downregulated
AAGCACA MIR218	347	-2.26	<0.010	Downregulated

Name: Gene set name

Size: number of genes in set

NES: Normalized enrichment score

FDR q-val: q value corrected for multiple hypothesis testing

AA vs EA: direction of change in gene set representation in AA tumors as compared to EA tumors
