# Supplementary material for: Race-associated Molecular Changes in Gynecologic Malignancies
Source: Cancer Res Commun. 2022 Feb 17;2(2):99–109. doi: 10.1158/2767-9764.CRC-21-0018 (PMC9390975; doi:10.1158/2767-9764.CRC-21-0018)
Supplement: Supplemental Table 4 — Differentially expressed microRNAs in Pan-Gyn cohort EA vs AA [file crc-21-0018-s04.pdf]

Supplemental Table 4

micro RNA	logFC	adj.P.Val
hsa-let-7b-3p|MIMAT0004482	-1.13	5.53E-26
hsa-let-7d-3p|MIMAT0004484	-0.97	3.80E-14
hsa-miR-328-3p|MIMAT0000752	-0.96	8.61E-16
hsa-miR-675-3p|MIMAT0006790	-0.90	1.20E-09
hsa-miR-127-3p|MIMAT0000446	-0.86	1.03E-12
hsa-miR-149-5p|MIMAT0000450	-0.81	2.47E-11
hsa-let-7c-5p|MIMAT0000064	-0.80	1.17E-12
hsa-miR-99b-5p|MIMAT0000689	-0.80	1.16E-16
hsa-miR-92b-3p|MIMAT0003218	-0.79	1.29E-08
hsa-miR-378a-5p|MIMAT0000731	-0.78	1.09E-10
hsa-miR-107|MIMAT0000104	-0.78	7.41E-08
hsa-miR-146b-3p|MIMAT0004766	-0.76	2.00E-10
hsa-let-7e-3p|MIMAT0004485	-0.76	7.92E-13
hsa-let-7b-5p|MIMAT0000063	-0.74	3.20E-18
hsa-miR-197-3p|MIMAT0000227	-0.72	1.20E-09
hsa-miR-125b-5p|MIMAT0000423	-0.72	5.21E-16
hsa-miR-125b-2-3p|MIMAT0004603	-0.71	7.95E-09
hsa-miR-134-5p|MIMAT0000447	-0.69	1.97E-13
hsa-miR-744-5p|MIMAT0004945	-0.67	2.42E-13
hsa-miR-574-3p|MIMAT0003239	-0.66	2.36E-17
hsa-miR-324-3p|MIMAT0000762	-0.65	3.47E-12
hsa-miR-205-5p|MIMAT0000266	-0.63	7.28E-04
hsa-miR-125a-5p|MIMAT0000443	-0.63	1.01E-13
hsa-miR-532-3p|MIMAT0004780	-0.63	5.18E-08
hsa-miR-331-3p|MIMAT0000760	-0.61	1.13E-17
hsa-miR-99b-3p|MIMAT0004678	-0.57	7.41E-08
hsa-miR-654-3p|MIMAT0004814	-0.54	1.25E-09
hsa-miR-625-3p|MIMAT0004808	-0.54	1.30E-07
hsa-miR-200b-5p|MIMAT0004571	-0.54	1.01E-07
hsa-miR-193a-5p|MIMAT0004614	-0.52	1.84E-08
hsa-miR-874-3p|MIMAT0004911	-0.52	2.08E-09
hsa-miR-486-5p|MIMAT0002177	-0.52	9.88E-05
hsa-miR-501-3p|MIMAT0004774	-0.52	1.03E-07
hsa-let-7i-3p|MIMAT0004585	-0.51	7.32E-12
hsa-miR-652-3p|MIMAT0003322	-0.51	9.81E-09

hsa-miR-194-5p|MIMAT0000460	0.53	1.03E-07
hsa-miR-93-5p|MIMAT0000093	0.53	3.27E-20
hsa-miR-28-5p|MIMAT0000085	0.54	4.42E-14
hsa-miR-103a-3p|MIMAT0000101	0.54	3.02E-21
hsa-miR-16-5p|MIMAT0000069	0.55	7.68E-09
hsa-let-7f-5p|MIMAT0000067	0.56	4.34E-09
hsa-miR-181a-3p|MIMAT0000270	0.61	1.06E-12
hsa-miR-452-5p|MIMAT0001635	0.64	1.72E-06
hsa-miR-30b-5p|MIMAT0000420	0.64	4.99E-10
hsa-miR-22-3p|MIMAT0000077	0.66	5.47E-10
hsa-miR-218-5p|MIMAT0000275	0.67	7.68E-09
hsa-miR-182-5p|MIMAT0000259	0.67	8.45E-08
hsa-miR-148a-3p|MIMAT0000243	0.67	4.74E-07
hsa-miR-98-5p|MIMAT0000096	0.70	1.92E-13
hsa-let-7a-3p|MIMAT0004481	0.73	2.46E-09
hsa-miR-196b-5p|MIMAT0001080	0.75	2.19E-04
hsa-miR-141-5p|MIMAT0004598	0.78	1.12E-10
hsa-miR-17-3p|MIMAT0000071	0.80	2.56E-31
hsa-miR-374b-5p|MIMAT0004955	0.81	1.37E-15
hsa-miR-185-5p|MIMAT0000455	0.82	7.58E-22
hsa-miR-199b-5p|MIMAT0000263	0.84	6.98E-07
hsa-miR-451a|MIMAT0001631	0.85	4.04E-09
hsa-miR-424-5p|MIMAT0001341	0.87	6.25E-07
hsa-miR-17-5p|MIMAT0000070	0.88	3.42E-24
hsa-miR-30a-5p|MIMAT0000087	0.90	1.16E-11
hsa-miR-582-3p|MIMAT0004797	0.90	1.31E-07
hsa-miR-126-3p|MIMAT0000445	0.92	3.38E-11
hsa-miR-140-5p|MIMAT0000431	0.95	1.12E-12
hsa-miR-200a-3p|MIMAT0000682	1.01	1.04E-14
hsa-miR-30e-5p|MIMAT0000692	1.02	2.67E-14
hsa-miR-15a-5p|MIMAT0000068	1.04	2.81E-19
hsa-miR-106b-5p|MIMAT0000680	1.07	4.64E-21
hsa-miR-708-3p|MIMAT0004927	1.12	1.53E-12
hsa-miR-29c-3p|MIMAT0000681	1.13	5.62E-13
hsa-miR-429|MIMAT0001536	1.18	7.96E-13
hsa-miR-338-3p|MIMAT0000763	1.23	1.16E-16
hsa-miR-142-5p|MIMAT0000433	1.26	7.41E-12
hsa-miR-660-5p|MIMAT0003338	1.28	1.88E-32

hsa-miR-374a-5p|MIMAT0000727	1.32	1.36E-17
hsa-miR-2355-5p|MIMAT0016895	1.33	2.13E-14
hsa-miR-126-5p|MIMAT0000444	1.37	5.23E-12
hsa-miR-32-5p|MIMAT0000090	1.38	3.69E-18
hsa-miR-101-3p|MIMAT0000099	1.39	7.03E-13
hsa-miR-21-5p|MIMAT0000076	1.42	1.03E-14
hsa-miR-141-3p|MIMAT0000432	1.43	2.09E-17
hsa-miR-29b-3p|MIMAT0000100	1.44	1.72E-15
hsa-miR-542-3p|MIMAT0003389	1.50	1.62E-13
hsa-miR-20a-5p|MIMAT0000075	1.58	9.31E-33
hsa-miR-142-3p|MIMAT0000434	1.63	3.67E-10
hsa-miR-9-5p|MIMAT0000441	1.65	3.70E-14
hsa-miR-19b-3p|MIMAT0000074	1.83	3.44E-19
hsa-miR-374a-3p|MIMAT0004688	2.13	1.05E-18

MicroRNA: full microRNA name

LogFC AA vs EA; Log2 (mean EA tumor RPKM) / (mean AA tumor RPKM) for each miR

adj.P.Val: P value adjusted for multiple hypothesis testing with BH method
