# Supplementary material for: Race-associated Molecular Changes in Gynecologic Malignancies
Source: Cancer Res Commun. 2022 Feb 17;2(2):99–109. doi: 10.1158/2767-9764.CRC-21-0018 (PMC9390975; doi:10.1158/2767-9764.CRC-21-0018)
Supplement: Supplemental Table 5 — Differentially methylated probes in Pan-Gyn cohort [file crc-21-0018-s05.pdf]

Supplementary Table 5

Name	Islands Name	Relation to Island	UCSC RefGene Name	UCSC RefGene Accession	UCSC RefGene Group	adj.P.Val	∆Beta
cg26131019	chr3:66550318-66551848	Island	LRIG1	NM 015541	1stExon	6.22E-167	-0.22
cg08634464	chr19:2900329-2901203	Island	ZNF57	NM 173480	Body	3.87E-38	-0.33
cg09039163	chr2:239009072-239009361	N Shore	ESPNL;ESPNL	NM 194312; NM 194312	1stExon; 5'UTR	3.12E-25	-0.12
cg22730830	chr16:2867245-2868002	Island	PRSS21; PRSS21; PRSS21	NM 144956; NM 006799; NM 144957	Body;Body;Body	4.61E-22	0.23
cg26987645		OpenSea	FMOD	NM 002023	TSS200	3.85E-21	0.12
cg17518825	chr2:46523789-46527140	N Shore	EPAS1	NM 001430	TSS1500	1.31E-20	-0.13
cg14417329	chr17:80186135-80189990	Island	SLC16A3;SLC16A3	NM 001042422;NM 004207	TSS200; TSS1500	2.04E-17	-0.12
cg01333788		OpenSea	GJB5	NM 005268	5'UTR	3.12E-17	-0.11
cg17904739	chr12:3862068-3862606	N Shore	EFCAB4B;EFCAB4B;EFCAB4B	NM 001144959;NM 032680; NM 001144958	5'UTR;5'UTR;5'UTR	4.70E-16	0.17
cg23817637		OpenSea	CLRN3	NM 152311	TSS1500	5.56E-16	-0.13
cg12854483		OpenSea	CNN1;CNN1	NM 001299; NM 001299	1stExon; 5'UTR	1.30E-11	0.14
cg02022375		OpenSea	KRTAP1-1	NM 030967	TSS200	1.38E-11	-0.12
cg05342835		OpenSea	SYNC;SYNC	NM 030786;	Body;Body	1.60E-11	0.14

				NM 001161708			
cg22487322	chr6:137365299-137366126	S Shore	IL20RA	NM 014432	TSS200	8.21E-10	0.13
cg15873301	chr3:12045652-12046627	N Shore	SYN2;SYN2	NM 133625; NM 003178	TSS1500;TSS1500	1.18E-08	-0.19
cg24512973	chr1:155163434-155163818	N Shore	MUC1;MUC1;MUC1;MUC1;MUC1;MUC1;MUC1	NM 001044391;NM 001044393;NM 001018016;NM 001044390;NM 001044392;NM 002456; NM 001018017	Body;Body;Body;Body;Body;Body;Body	2.93E-08	0.15
cg16731240	chr19:52390841-52391368	Island	ZNF577; ZNF577; ZNF577	NR 024181; NM 032679; NM 001135590	TSS200; TSS200; TSS200	6.36E-08	0.15
cg18182399	chr2:220283200-220283750	N Shore	DES;DES	NM 001927; NM 001927	1stExon; 5'UTR	2.36E-07	0.27
cg18738906		OpenSea	SCNN1A;SCNN1A;SCNN1A	NM 001159575;NM 001038; NM 001159576	Body;Body;1stExon	4.30E-07	0.20
cg22506059	chr22:37914768-37915883	S Shore	CARD10	NM 014550	TSS1500	1.04E-06	0.14
cg06818777	chr17:48545570-48546900	Island	CHAD;ACSF2	NM 001267; NM 025149	TSS1500;Body	3.94E-05	0.12

cg06958829	chr17:48545570-48546900	Island	ACSF2;CHAD	NM 025149;NM 001267	Body;1st Exon	4.58E-05	0.24
cg15158783	chr4:74847528-74847830	Island	PF4	NM 002619	1stExon	5.24E-05	0.13
cg00333528		OpenSea	GABRR1;GABRR1	NM 002042;NM 002042	5'UTR;1stExon	5.62E-05	0.12
cg01081263	chr11:9112461-9113459	Island	SCUBE2;SCUBE2	NM 020974;NM 001170690	Body;Body	7.14E-05	0.12
cg27278294	chrX:149736720-149737756	N Shore	MTM1	NM 000252	TSS1500	7.38E-05	-0.10
cg19884600		OpenSea	PYHIN1;PYHIN1;PYHIN1;PYHIN1	NM 198930;NM 198928;NM 198929;NM 152501	5'UTR;5'UTR;5'UTR;5'UTR	2.72E-04	-0.11
cg14925024	chr1:228289669-228291184	S Shore	C1orf35	NM 024319	TSS1500	2.94E-04	-0.12
cg08899626		OpenSea	LDB2;LDB2;LDB2;LDB2	NM 001290;NM 001130834;NM 001130834;NM 001290	1stExon;1stExon;5'UTR;5'UTR	5.63E-04	0.11
cg25593948	chr6:50787286-50788091	N Shore	TFAP2B	NM 003221	1stExon	7.69E-04	0.10
cg05942574	chr17:48636103-48639279	Island	CACNA1G;CACNA1G;CACNA1G;CACNA1G;CACNA1G;CACNA1G;CACN	NM 198384;NM 198397;NM 198396;NM 198379;NM 198383;	TSS1500;TSS1500;TSS1500;TSS1500;TSS1500;TSS1500;TSS1500;TSS1500;TSS150	7.96E-04	0.13

			A1G;CACNA1G;CACNA1G;CACNA1G;CACNA1G;CACNA1G	NM 198380;NM 198387;NM 198378;NM 198377;NM 198386;NM 198376;NM 198385;NM 018896;NM 198388;NM 198382	0;TSS1500;TSS1500;TSS1500;TSS1500;TSS1500;TSS1500		
cg16415058	chr10:108923780-108924805	Island	SORCS1;SORCS1	NM 001013031;NM 052918	1stExon;1stExon	8.34E-04	0.19
cg20773127	chr4:111397677-111398089	N Shore	ENPEP	NM 001977	TSS200	8.40E-04	0.11
cg10014293	chr16:56701863-56702208	S Shore	MT1H	NM 005951	Body	9.56E-04	0.12
cg22605643		OpenSea	RGS4;RGS4;RGS4;RGS4	NM 005613;NM 001102445;NM 001113381;NM 005613	1stExon;Body;TSS200;5'UTR	1.10E-03	0.10
cg03760483	chr17:6898820-6900427	Island	ALOX12	NM 000697	TSS200	1.26E-03	0.12
cg10925082		OpenSea	ARHGDIB	NM 001175	TSS200	1.52E-03	0.11
cg17142183		OpenSea	IL1R2	NM 004633	TSS200	1.61E-03	-0.11
cg25438963	chr6:26045644-26046033	Island	HIST1H3C	NM 003531	1stExon	3.08E-03	0.26
cg15799267		OpenSea	ALOX15B;ALOX15B;ALOX	NM 001141;NM	5'UTR;5'UTR;1stExon;5'U	3.76E-03	0.11

			15B;ALOX15B;ALOX15B;ALOX15B	001039130;NM 001039131;NM 001039131;NM 001141;NM 001039130	TR;1stExon;1stExon		
cg09137696	chr16:56671937-56672530	Island	MT1A	NM 005946	TSS200	4.59E-03	0.17
cg21922574	chr18:70533965-70536871	Island	NETO1	NM 138966	TSS1500	8.03E-03	0.17
cg07636178	chr6:26045644-26046033	N Shore	HIST1H3C	NM 003531	TSS200	9.43E-03	0.11
cg09313705	chr17:46620367-46621373	S Shore	HOXB2	NM 002145	TSS200	9.58E-03	0.12
cg03573747		OpenSea	ADIPOQ	NM 004797	5'UTR	1.18E-02	0.11
cg09980522	chr11:105481126-105481422	S Shore	GRIA4;GRIA4;GRIA4;GRIA4	NM 001077243;NM 001077244;NM 001112812;NM 000829	Body;Body;1stExon;Body	1.34E-02	0.14
cg02916816	chr14:24044886-24046760	S Shore	JPH4;JPH4	NM 001146028;NM 032452	5'UTR;5'UTR	1.39E-02	0.14
cg20449692	chr3:170136242-170137886	Island	CLDN11	NM 005602	1stExon	1.43E-02	0.12
cg11762346		OpenSea	HKDC1;HKDC1	NM 025130;NM 025130	5'UTR;1stExon	1.72E-02	0.18
cg15740508		OpenSea	AMPD1	NM 000036	TSS1500	1.84E-02	0.16
cg26029902	chr20:56884738-56885222	N Shore	RAB22A;PPP4R1L	NM 020673;NR 003505	TSS1500;Body	1.89E-02	0.13

cg25239996	chr20:44746822-44747060	N Shore	CD40;CD40	NM 152854; NM 001250	TSS200; TSS200	1.97E-02	0.12
cg23995753	chr2:160760604-160761452	Island	LY75	NM 002349	Body	2.01E-02	0.15
cg12100791	chr16:31213566-31214287	S Shore	PYCARD;PYCARD	NM 013258; NM 145182	TSS200; TSS200	2.02E-02	0.10
cg08124030		OpenSea	TM4SF1	NM 014220	1stExon	2.14E-02	0.10
cg21880903		OpenSea	KLB	NM 175737	1stExon	2.19E-02	0.14
cg19994834	chrX:122318752-122319149	N Shore	GRIA3;GRIA3	NM 007325; NM 000828	TSS200; TSS200	2.33E-02	0.10
cg01058368		OpenSea	CDH10	NM 006727	TSS1500	2.83E-02	0.11
cg26385222	chr7:150496809-150498206	Island	TMEM176B;TMEM176B;TMEM176A;TMEM176B;TMEM176B;TMEM176B	NM 001101313;NM 001101311;NM 018487; NM 014020; NM 001101312;NM 001101314	TSS1500;5'UTR;TSS1500; 5'UTR;5'UTR;5'UTR	3.24E-02	0.10
cg00891278	chr3:126113547-126113967	Island	CCDC37; CCDC37	NM 182628; NM 182628	1stExon; 5'UTR	3.73E-02	0.13
cg15077070	chr2:102803672-102804556	N Shore	IL1RL2	NM 003854	TSS200	4.53E-02	0.11

Name: DNA methylation probe name
Relations to Island: position of methylation probe relative to nearest island
UCSC RefGene Name
UCSC RefGene Accession number
UCSC RefGeneGroup: Assigned DNA loci per UCSC designation
adj.P.Val: P-value adjusted for multiple hypothesis testing
∆ Beta: Difference in mean beta methylation level (EA – AA)
