# Supplementary material for: Race-associated Molecular Changes in Gynecologic Malignancies
Source: Cancer Res Commun. 2022 Feb 17;2(2):99–109. doi: 10.1158/2767-9764.CRC-21-0018 (PMC9390975; doi:10.1158/2767-9764.CRC-21-0018)
Supplement: Supplemental Table 6 — Differentially methylated probes analyzed by tumor site [file crc-21-0018-s06.pdf]

Supplementary Table 6

chr	pos	strand	Name	UCSC RefGene Name	adj.P.Val	∆Beta	Tumor
chrX	151306718	+	cg04950711	MAGEA10;MAGEA10	0.02	-0.29	OV
chr7	100544704	-	cg00644033		0.01	-0.28	OV
chrX	52840736	-	cg25993152	XAGE5	0.02	-0.27	OV
chr10	44881551	+	cg00499822	CXCL12;CXCL12;CXCL12	0.00	-0.21	OV
chr19	2901147	-	cg08634464	ZNF57	0.01	-0.20	OV
chr6	41130718	+	cg20095587	TREM2	0.05	-0.19	OV
chr9	117905075	+	cg04761824	1-Dec	0.01	-0.18	OV
chr19	42260485	-	cg26813458	CEACAM6	0.02	-0.17	OV
chr7	99573278	+	cg19465374	AZGP1	0.03	-0.17	OV
chrX	151307053	-	cg19964192	MAGEA10;MAGEA10	0.00	-0.16	OV
chr14	96152109	+	cg27504299	TCL1B;TCL1B	0.02	-0.16	OV
chr20	57427830	+	cg17414107	GNAS;GNAS;GNAS	0.02	-0.16	OV
chr1	159258877	-	cg14696870	FCER1A	0.04	-0.16	OV
chr19	1512314	-	cg04601137	ADAMTSL5	0.02	-0.15	OV
chr5	54281336	-	cg20451680	ESM1;ESM1	0.03	-0.15	OV
chr4	69111580	+	cg19510180	TMPRSS11B	0.02	-0.15	OV
chr6	32731327	-	cg04345908	HLA-DQB2	0.03	-0.15	OV
chr2	95537475	-	cg05723825	TEKT4	0.00	-0.15	OV
chrX	118370356	-	cg07876586	PGRMC1	0.00	-0.15	OV
chr20	57427738	+	cg27661264	GNAS;GNAS;GNAS	0.03	-0.14	OV
chr3	66550735	+	cg26131019	LRIG1	0.00	-0.14	OV
chr6	110011156	-	cg01500097	AKD1;FIG4;AKD1	0.05	-0.13	OV
chr17	39197721	-	cg02022375	KRTAP1-1	0.01	-0.13	OV
chr11	55703443	-	cg25890048	OR5I1	0.01	-0.12	OV
chr19	10736038	-	cg17826679	SLC44A2;SLC44A2	0.03	-0.12	OV

chr6	109760831	+	cg20811607	PPIL6;PPIL6;SMPD2	0.01	-0.11	OV
chr1	203059178	-	cg13626881		0.04	-0.11	OV
chr21	45705686	-	cg17356252	AIRE	0.02	-0.11	OV
chrX	100740937	+	cg14457691	ARMCX4	0.05	-0.10	OV
chr1	35220814	-	cg01333788	GJB5	0.02	-0.10	OV
chr2	239009036	-	cg09039163	ESPNL;ESPNL	0.02	-0.10	OV
chrX	69487197	-	cg11494656	ARR3	0.04	-0.10	OV
chrX	41783155	-	cg16269097	CASK;CASK;CASK	0.03	-0.10	OV
chrX	152086723	-	cg16022279	ZNF185	0.01	-0.10	OV
chrX	153448957	+	cg06325687	OPN1MW2;OPN1MW	0.03	-0.10	OV
chr21	36421472	+	cg04915566	RUNX1;RUNX1	0.01	0.11	OV
chr12	15373987	-	cg19205533	RERG	0.03	0.14	OV
chr11	85780971	+	cg01120308	PICALM;PICALM	0.03	0.14	OV
chr16	56642024	+	cg17886959	MT2A	0.01	0.14	OV
chr3	111852156	+	cg25462303	GCET2;GCET2	0.02	0.14	OV
chr1	163039012	-	cg22605643	RGS4;RGS4;RGS4;RGS4	0.04	0.17	OV
chr12	15114703	-	cg10925082	ARHGDIB	0.03	0.20	OV
chr14	39735211	+	cg13277939	CTAGE5;CTAGE5;CTAGE5;CTAGE5	0.04	0.23	OV
chr19	49249932	-	cg10234985	IZUMO1;IZUMO1	0.05	0.26	OV
chr19	2901147	-	cg08634464	ZNF57	5.30E-20	-0.34	BRCA
chr11	65405362	-	cg25361844	SIPA1	6.79E-05	-0.29	BRCA
chr3	139258939	+	cg23363832	RBP1;RBP1;RBP1	1.22E-04	-0.25	BRCA
chr8	23083353	-	cg26530341	TNFRSF10A	5.20E-10	-0.24	BRCA
chr5	118691033	-	cg07086380	TNFAIP8;TNFAIP8	1.06E-08	-0.23	BRCA
chr3	66550735	+	Cg 26131019	LRIG1	4.62E-88	-0.22	BRCA
chr15	48936953	-	cg18671950	FBN1	2.53E-02	-0.22	BRCA

chr9	90113813	-	cg15746719	DAPK1	9.82E-08	-0.21	BRCA
chr2	202098951	-	cg26799474	CASP8;CASP8;CASP8;CASP8	5.35E-04	-0.19	BRCA
chr8	109799756	+	cg15042080	TMEM74;TMEM74	5.17E-03	-0.19	BRCA
chr3	142838847	-	cg00995327	CHST2;CHST2	2.95E-02	-0.17	BRCA
chr11	44330903	+	cg26365854	ALX4	3.26E-03	-0.16	BRCA
chr17	33701321	-	cg18108623	SLFN11;SLFN11;SLFN11;SLFN11;SLFN11	3.45E-02	-0.16	BRCA
chr10	64575798	-	cg19355190	EGR2;EGR2;EGR2;EGR2;EGR2	1.25E-02	-0.16	BRCA
chr16	1430216	-	cg11911951	UNKL	4.38E-04	-0.16	BRCA
chr17	25798973	-	cg05246522	KSR1	2.42E-02	-0.15	BRCA
chr3	49378032	+	cg18886444	USP4;USP4	1.29E-05	-0.15	BRCA
chr11	44327399	-	cg04970352	ALX4	1.18E-02	-0.14	BRCA
chr10	129691429	+	cg23817637	CLRN3	4.08E-17	-0.14	BRCA
chr14	85996495	+	cg17410236	FLRT2;FLRT2	2.44E-02	-0.14	BRCA
chr15	101419074	-	cg23191950	ALDH1A3	5.06E-06	-0.14	BRCA
chr11	65405903	-	cg26668713	SIPA1	6.72E-06	-0.14	BRCA
chr4	48485233	-	cg19283196	SLC10A4	3.37E-02	-0.14	BRCA
chr8	133772889	+	cg20955688	TMEM71;TMEM71;TMEM71;TMEM71	3.05E-04	-0.13	BRCA
chr20	56286697	+	cg00138126	PMEPA1;PMEPA1;PMEPA1	2.68E-04	-0.13	BRCA
chr19	33863876	-	cg15046693	CEBPG	1.00E-05	-0.13	BRCA
chr10	82049429	+	cg19423196	MAT1A;MAT1A	4.97E-03	-0.13	BRCA
chr3	12045459	-	cg15873301	SYN2;SYN2	8.05E-05	-0.13	BRCA
chr5	76114373	-	cg24573501	F2RL1	2.52E-03	-0.13	BRCA
chr2	239009036	-	cg09039163	ESPNL;ESPNL	5.30E-20	-0.13	BRCA
chr17	80186273	-	cg14417329	SLC16A3;SLC16A3	3.20E-12	-0.13	BRCA
chr7	49813763	-	cg00333226	VWC2	6.08E-03	-0.13	BRCA
chr19	6739192	-	cg02085507	TRIP10	1.85E-02	-0.13	BRCA

chr8	82192606	+	cg19904463	FABP5	9.78E-06	-0.13	BRCA
chr1	25255838	+	cg00117172	RUNX3;RUNX3	2.27E-03	-0.13	BRCA
chr8	125984927	+	cg23968383	ZNF572	3.71E-08	-0.13	BRCA
chr9	36037454	+	cg12717594	RECK	3.69E-03	-0.12	BRCA
chr17	46800639	-	cg01543654	C17orf93;PRAC	2.07E-02	-0.12	BRCA
chr2	46523461	-	cg17518825	EPAS1	2.60E-14	-0.12	BRCA
chr2	43019614	-	cg20289949	HAAO	1.52E-02	-0.12	BRCA
chr19	35981677	-	cg10305797	KRTDAP	1.77E-05	-0.12	BRCA
chr13	78492916	-	cg23316360	EDNRB;EDNRB;EDNRB;EDNRB;EDNRB	9.00E-03	-0.12	BRCA
chr14	76045348	+	cg04001333	FLVCR2	1.32E-08	-0.12	BRCA
chr18	2847309	+	cg09009111	EMILIN2	1.73E-02	-0.12	BRCA
chr17	27369780	+	cg06144905	PIPOX	6.44E-03	-0.12	BRCA
chr6	110797497	+	cg22334665	SLC22A16	4.34E-03	-0.12	BRCA
chr9	34263021	+	cg19947621	KIF24	1.77E-04	-0.12	BRCA
chr1	76540465	-	cg12601757	ST6GALNAC3; ST6GALNAC3; ST6GALNAC3; ST6GALNAC3	3.40E-02	-0.12	BRCA
chr9	90114156	-	cg24754277	DAPK1	6.56E-04	-0.12	BRCA
chr8	27850178	+	cg07634191	SCARA5;SCARA5	1.78E-05	-0.12	BRCA
chr7	23510648	-	cg02860543	IGF2BP3	3.39E-02	-0.11	BRCA
chr3	139258822	+	cg06543018	RBP1;RBP1;RBP1	9.24E-08	-0.11	BRCA
chr6	151772946	-	cg00510787	C6orf211;RMND1	2.60E-14	-0.11	BRCA
chr2	10830764	-	cg02196655	NOL10	1.42E-04	-0.11	BRCA
chr17	39197721	-	cg02022375	KRTAP1-1	5.68E-08	-0.11	BRCA
chr3	71834640	+	cg08555612	PROK2;PROK2	6.97E-03	-0.11	BRCA
chr1	158799935	-	cg25119415	MNDA	6.20E-03	-0.11	BRCA
chr17	27944585	-	cg06038133	CORO6	1.79E-03	-0.11	BRCA
chr12	112452424	-	cg26789453	TMEM116;ERP29;ERP29	3.34E-03	-0.11	BRCA

chr15	101419017	-	cg19224278	ALDH1A3	4.36E-04	-0.11	BRCA
chr20	53092781	+	cg11822659	DOK5	4.40E-06	-0.11	BRCA
chr8	133772742	+	cg27159719	TMEM71;TMEM71;TMEM71;TMEM71	2.10E-03	-0.11	BRCA
chr19	39687963	+	cg19917856	NCCRP1	1.03E-02	-0.11	BRCA
chr1	234040833	-	cg25437385	SLC35F3	4.24E-02	-0.11	BRCA
chr3	148939523	-	cg17439694	CP	2.96E-11	-0.11	BRCA
chr1	203156246	-	cg07423149	CHI3L1	1.22E-02	-0.11	BRCA
chr4	111557894	-	cg05522383	PITX2;PITX2	9.50E-03	-0.11	BRCA
chr12	75728469	-	cg21087137	GLIPR1L1;GLIPR1L1	6.41E-03	-0.10	BRCA
chr17	10102558	-	cg13991233	GAS7	2.30E-03	-0.10	BRCA
chr8	41165699	-	cg02388150	SFRP1	6.32E-04	-0.10	BRCA
chr3	44902933	-	cg16615211	MIR564;TMEM42	1.90E-02	-0.10	BRCA
chr17	6616653	-	cg16652063	SLC13A5;SLC13A5;SLC13A5;SLC13A5	2.89E-02	-0.10	BRCA
chr1	205819179	+	cg14159672	PM20D1	8.63E-11	-0.10	BRCA
chr1	171810972	-	cg23391785	DNM3;DNM3	6.27E-04	-0.10	BRCA
chr18	56530302	+	cg12406559	ZNF532;ZNF532	3.21E-02	-0.10	BRCA
chr13	47469654	+	cg00308665	HTR2A;HTR2A	1.49E-03	0.10	BRCA
chr19	663973	+	cg03605761	RNF126	1.39E-06	0.10	BRCA
chr8	25281609	+	cg22502171	GNRH1;GNRH1;GNRH1	1.98E-06	0.10	BRCA
chr16	30406122	+	cg16014085	ZNF48	2.45E-03	0.10	BRCA
chr19	1492043	-	cg02674804	REEP6	5.63E-05	0.10	BRCA
chr3	122334227	-	cg22468055	PARP15;PARP15	1.65E-05	0.10	BRCA
chr6	29455532	+	cg01078434	MAS1L	3.47E-02	0.10	BRCA
chr16	15527940	-	cg07977490	C16orf45	3.87E-09	0.10	BRCA
chr4	26493496	-	cg04353483	CCKAR	1.77E-02	0.10	BRCA
chr18	48189538	-	cg26946769	MAPK4	5.74E-03	0.10	BRCA

chr18	63417392	+	cg04564030	CDH7;CDH7	2.87E-02	0.11	BRCA
chr22	37916157	+	cg22506059	CARD10	3.19E-08	0.11	BRCA
chr17	46622491	-	cg09313705	HOXB2	2.21E-04	0.11	BRCA
chr19	58262242	-	cg02682905	ZNF776	6.63E-04	0.11	BRCA
chr20	43935361	+	cg21835643	MATN4;MATN4;RBPJL;MATN4	3.59E-02	0.11	BRCA
chr1	4715520	-	cg17525406	AJAP1;AJAP1	3.61E-02	0.11	BRCA
chr20	44657948	+	cg22752533	SLC12A5;SLC12A5;SLC12A5	1.29E-03	0.11	BRCA
chr8	80523461	-	cg23326689	STMN2	4.31E-06	0.11	BRCA
chr7	64126140	+	cg12856392	ZNF107;ZNF107	5.26E-05	0.11	BRCA
chr1	203320223	-	cg03764585	FMOD;FMOD	3.40E-06	0.11	BRCA
chr4	5895098	+	cg22680204	CRMP1	1.44E-02	0.11	BRCA
chr17	66950545	-	cg21660392	ABCA8	2.92E-03	0.11	BRCA
chr3	112360952	+	cg21307628	CCDC80;CCDC80	2.72E-04	0.11	BRCA
chr7	89840731	+	cg27626102	STEAP2;STEAP2	4.71E-05	0.11	BRCA
chr5	24645487	-	cg01058368	CDH10	1.24E-06	0.11	BRCA
chr3	187388225	+	cg13206017	SST	1.48E-03	0.11	BRCA
chr5	36301436	-	cg26511075	RANBP3L;RANBP3L	2.37E-04	0.11	BRCA
chr6	31088188	+	cg08424423	CDSN;PSORS1C1	7.08E-06	0.11	BRCA
chr2	182544413	-	cg02836529	NEUROD1	5.08E-04	0.11	BRCA
chr20	1538671	-	cg25737664	SIRPD	1.89E-02	0.11	BRCA
chr16	6068835	-	cg19378133	A2BP1;A2BP1	2.18E-03	0.11	BRCA
chr1	151009588	-	cg11584936	BNIPL;BNIPL	5.26E-03	0.11	BRCA
chr1	153283994	-	cg06275635	PGLYRP3	1.73E-02	0.11	BRCA
chr16	31214417	-	cg12100791	PYCARD;PYCARD	1.45E-03	0.11	BRCA
chr10	17171695	-	cg10707565	CUBN	2.27E-03	0.11	BRCA
chr4	16900199	-	cg08899626	LDB2;LDB2;LDB2;LDB2	4.64E-03	0.11	BRCA
chr4	683240	+	cg17952262	MFSD7	9.54E-05	0.12	BRCA

chr3	186560617	-	cg03573747	ADIPOQ	4.38E-05	0.12	BRCA
chr1	33160791	-	cg05342835	SYNC;SYNC	6.32E-11	0.12	BRCA
chr1	104160257	+	cg22215192	AMY2A	4.85E-06	0.12	BRCA
chr3	8693706	+	cg22959932	C3orf32;C3orf32	2.16E-05	0.12	BRCA
chr3	58523313	+	cg13705284	ACOX2	1.80E-08	0.12	BRCA
chr6	167275395	-	cg13217373	RPS6KA2	7.81E-07	0.12	BRCA
chr8	6420506	-	cg24670715	ANGPT2;ANGPT2;ANGPT2;ANGPT2;ANGPT2;ANGPT2;MCPH1	5.15E-05	0.12	BRCA
chr20	39928631	-	cg22294908	ZHX3;ZHX3	1.16E-02	0.12	BRCA
chr1	206317403	+	cg21478437	CTSE;CTSE	1.23E-04	0.12	BRCA
chr5	140588299	+	cg07899016	PCDHB12;PCDHB12	7.69E-04	0.12	BRCA
chr1	11867308	-	cg14472778	CLCN6;CLCN6;CLCN6;CLCN6;MTHFR	1.48E-05	0.12	BRCA
chr9	104248926	+	cg06912252	C9orf125	5.46E-03	0.12	BRCA
chr17	3600461	+	cg13246592	P2RX5;P2RX5;P2RX5	3.48E-04	0.12	BRCA
chr11	57148215	-	cg24459209	PRG3	1.20E-05	0.12	BRCA
chr10	8094679	+	cg04765277	FLJ45983;FLJ45983	3.33E-03	0.12	BRCA
chr12	49449136	+	cg13007988	MLL2	1.71E-03	0.12	BRCA
chr12	11463844	+	cg22692158	PRB4	4.19E-06	0.12	BRCA
chr7	114055419	+	cg05232889	FOXP2;FOXP2;FOXP2	4.55E-04	0.12	BRCA
chr6	30659643	+	cg16979445	NRM	1.62E-03	0.12	BRCA
chr11	6462452	+	cg11547724	HPX	8.66E-07	0.12	BRCA
chr6	166722773	+	cg05089968	PRR18	2.93E-05	0.12	BRCA
chr5	9546755	-	cg03702236	SEMA5A	7.33E-06	0.12	BRCA
chr12	21525662	-	cg15583072	SLCO1A2;IAPP	9.54E-05	0.12	BRCA
chr1	183622007	-	cg20579480	APOBEC4;RGL1	1.46E-05	0.12	BRCA
chr14	23653098	+	cg27035169	SLC7A8	1.30E-11	0.12	BRCA

chr11	63272554	+	cg11484576	LGALS12;LGALS12;LGALS12	1.93E-06	0.13	BRCA
chr7	87855714	+	cg02399455	SRI	1.45E-03	0.13	BRCA
chr20	58507171	+	cg07347645	SYCP2;SYCP2	2.87E-02	0.13	BRCA
chr1	2985218	+	cg08528984	FLJ42875;PRDM16;PRDM16;FLJ42875	8.57E-03	0.13	BRCA
chr17	48546118	-	cg06958829	ACSF2;CHAD	2.60E-08	0.13	BRCA
chr8	144120106	-	cg07770222	C8orf31	1.52E-03	0.13	BRCA
chr12	53208762	+	cg12610744	KRT4	5.06E-06	0.13	BRCA
chr2	167343774	+	cg05331214	SCN7A	3.49E-03	0.13	BRCA
chr1	155161784	-	cg24512973	MUC1;MUC1;MUC1;MUC1;MUC1;MUC1;MUC1	5.45E-09	0.13	BRCA
chr2	121223909	-	cg17204557	LOC84931	1.06E-07	0.13	BRCA
chr8	132054555	-	cg13912117		8.58E-03	0.13	BRCA
chr1	197238398	+	cg00321478	CRB1	1.55E-04	0.13	BRCA
chr22	42896688	+	cg12078929	SERHL	4.76E-07	0.13	BRCA
chr17	7942406	-	cg15799267	ALOX15B;ALOX15B;ALOX15B;ALOX15B;ALOX15B;ALOX15B	7.56E-04	0.13	BRCA
chr20	58533443	+	cg20895028	CDH26	1.88E-04	0.13	BRCA
chr2	220283175	+	cg18182399	DES;DES	7.15E-05	0.13	BRCA
chr3	113957903	-	cg03109316	ZNF80	1.83E-02	0.13	BRCA
chr2	119699682	-	cg11009736	MARCO	1.67E-05	0.14	BRCA
chr1	207039302	-	cg23282674	IL20	2.02E-04	0.14	BRCA
chr5	95769008	+	cg23187653	PCSK1	8.52E-04	0.14	BRCA
chr10	108923781	-	cg16415058	SORCS1;SORCS1	8.75E-08	0.14	BRCA
chr2	183731407	+	cg20308679	FRZB;FRZB	8.79E-06	0.14	BRCA
chr1	201617042	+	cg25167447	NAV1	9.82E-08	0.14	BRCA
chr17	48637104	-	cg05942574	CACNA1G;CACNA1G;CACNA1G;CACNA1G;CACNA1G;CACN	8.28E-05	0.14	BRCA

				A1G;CACNA1G;CACNA1G;CACNA1G;CACNA1G;CACNA1G;CACNA1G;CACNA1G;CACNA1G;CACNA1G			
chr1	226012913	-	cg24928687	EPHX1;EPHX1	7.86E-04	0.14	BRCA
chr20	58533495	-	cg24607535	CDH26;CDH26	1.57E-03	0.14	BRCA
chr19	15052824	-	cg03544379	OR7C2	3.09E-03	0.14	BRCA
chr1	16330784	-	cg24525573	C1orf64;C1orf64	9.69E-05	0.14	BRCA
chr14	99177777	-	cg06906435	C14orf177	6.04E-03	0.15	BRCA
chr10	8094641	-	cg00779924	FLJ45983;FLJ45983	1.96E-03	0.15	BRCA
chr17	3379567	+	cg02228185	ASPA;ASPA	9.39E-07	0.15	BRCA
chr1	153013830	-	cg14826683	SPRR2D	1.13E-03	0.15	BRCA
chr11	9112471	-	cg01081263	SCUBE2;SCUBE2	4.06E-09	0.15	BRCA
chr10	26223310	-	cg23771603	MYO3A	6.06E-03	0.15	BRCA
chr19	52391250	+	cg16731240	ZNF577;ZNF577;ZNF577	1.87E-06	0.15	BRCA
chr3	195310883	-	cg05624196	APOD;APOD	2.39E-05	0.16	BRCA
chr5	137071523	+	cg13847070	KLHL3;KLHL3	3.06E-05	0.16	BRCA
chr1	184944288	-	cg25182523	FAM129A	1.94E-07	0.16	BRCA
chr11	26743425	+	cg05953243	SLC5A12;SLC5A12	4.59E-04	0.16	BRCA
chr7	86973677	-	cg15350036	CROT;CROT;CROT;TP53TG1	5.74E-06	0.16	BRCA
chr6	56112291	-	cg13830624	COL21A1;COL21A1	2.35E-02	0.16	BRCA
chr1	203058954	-	cg11719784		2.99E-04	0.17	BRCA
chr18	55400167	-	cg18085435	ATP8B1	4.21E-05	0.17	BRCA
chr12	3861567	+	cg17904739	EFCAB4B;EFCAB4B;EFCAB4B	1.03E-09	0.17	BRCA
chr4	76555547	-	cg14988503	CDKL2;CDKL2	3.41E-02	0.17	BRCA
chr3	184095505	+	cg11819637	THPO	6.77E-07	0.17	BRCA

chr15	45803341	+	cg08057475	SLC30A4;C15orf21	5.84E-04	0.17	BRCA
chr2	157280411	-	cg24579667		4.93E-05	0.18	BRCA
chr1	173019720	-	cg19589427	TNFSF18	3.09E-06	0.18	BRCA
chr17	48546620	-	cg06818777	CHAD;ACSF2	5.86E-07	0.18	BRCA
chr11	26744467	-	cg20092728	SLC5A12	5.91E-03	0.18	BRCA
chr18	70535925	+	cg21922574	NETO1	2.66E-06	0.18	BRCA
chr6	50786670	+	cg25593948	TFAP2B	1.99E-04	0.18	BRCA
chr5	13944491	-	cg03503295	DNAH5	8.39E-06	0.18	BRCA
chr16	56672415	-	cg09137696	MT1A	1.20E-03	0.19	BRCA
chr4	141178469	+	cg04457051	SCOC;SCOC	3.04E-10	0.19	BRCA
chr12	11062035	-	cg08658594	TAS2R13;TAS2R13;PRR4;PRH1	7.08E-08	0.19	BRCA
chr6	137366322	+	cg22487322	IL20RA	4.97E-16	0.20	BRCA
chr16	2867434	-	cg22730830	PRSS21;PRSS21;PRSS21	2.26E-19	0.21	BRCA
chr1	203320386	-	cg26987645	FMOD	8.72E-14	0.22	BRCA
chr12	6483739	-	cg18738906	SCNN1A;SCNN1A;SCNN1A	9.84E-06	0.23	BRCA
chr17	49295615	-	cg02880679	MBTD1	3.04E-06	0.23	BRCA
chr1	248020812	-	cg07533148	TRIM58	1.24E-04	0.27	BRCA
chr1	168250628	+	cg17095936	TBX19	9.72E-08	0.35	BRCA
chr10	135044114	+	cg09053680	UTF1	0.03	-0.48	UCEC
chr6	73332073	-	cg24687051	KCNQ5;KCNQ5;KCNQ5;KCNQ5;KCNQ5	0.00	-0.48	UCEC
chr2	68546507	-	cg07080358	CNRIP1;CNRIP1	0.01	-0.45	UCEC
chr11	20690957	+	cg17371081	NELL1;NELL1	0.01	-0.43	UCEC
chr18	5543801	+	cg00027083	EPB41L3	0.03	-0.39	UCEC
chr18	49866548	+	cg21669679	DCC;DCC	0.01	-0.36	UCEC
chr4	165878091	-	cg00393585	C4orf39;TRIM61	0.01	-0.35	UCEC
chr12	85306916	+	cg03064067	SLC6A15;SLC6A15;SLC6A15	0.00	-0.34	UCEC

chr14	38677218	+	cg27590397	SSTR1;SSTR1	0.01	-0.33	UCEC
chr18	74962369	+	cg04534765	GALR1;GALR1	0.02	-0.32	UCEC
chr18	74961787	+	cg10486998	GALR1	0.03	-0.32	UCEC
chr14	27066634	-	cg18488855	NOVA1;NOVA1;NOVA1	0.02	-0.31	UCEC
chr5	160975024	+	cg01939681	GABRB2;GABRB2	0.01	-0.31	UCEC
chr19	2901147	-	cg08634464	ZNF57	0.00	-0.30	UCEC
chr10	1779667	+	cg05307923	ADARB2;ADARB2	0.01	-0.30	UCEC
chr16	85932853	-	cg24826867	IRF8	0.02	-0.30	UCEC
chr18	74961785	+	cg15343119	GALR1	0.03	-0.30	UCEC
chr2	21022565	-	cg09109450	C2orf43	0.00	-0.28	UCEC
chr18	63417392	+	cg04564030	CDH7;CDH7	0.04	-0.28	UCEC
chr4	66535145	+	cg18420965	EPHA5;EPHA5	0.01	-0.26	UCEC
chr19	46477338	+	cg21461100	NOVA2	0.01	-0.26	UCEC
chr18	74961727	+	cg26721264	GALR1	0.03	-0.26	UCEC
chr21	31312091	+	cg09542111	GRIK1;GRIK1;GRIK1;GRIK1	0.02	-0.26	UCEC
chr20	53092781	+	cg11822659	DOK5	0.01	-0.25	UCEC
chr2	45169447	+	cg13163729	SIX3	0.01	-0.25	UCEC
chr11	131780332	-	cg20881910	NTM;NTM;NTM;NTM	0.02	-0.25	UCEC
chr12	85306474	-	cg14449051	SLC6A15;SLC6A15;SLC6A15;SLC6A15;SLC6A15;SLC6A15	0.02	-0.25	UCEC
chr10	128076936	+	cg11668923	ADAM12;ADAM12;ADAM12;ADAM12	0.05	-0.25	UCEC
chr3	12045459	-	cg15873301	SYN2;SYN2	0.01	-0.24	UCEC
chr6	73331652	+	cg15717808	KCNQ5;KCNQ5;KCNQ5;KCNQ5;KCNQ5;KCNQ5;KCNQ5;KCNQ5;KCNQ5;KCNQ5	0.01	-0.24	UCEC
chr1	53528612	-	cg23092823	PODN	0.05	-0.23	UCEC
chr8	24815006	+	cg13266631	NEFL	0.02	-0.23	UCEC

chr5	45695922	-	cg06498267	HCN1	0.01	-0.22	UCEC
chr4	4228571	+	cg14882700	OTOP1	0.02	-0.22	UCEC
chr1	158901938	+	cg19884600	PYHIN1;PYHIN1;PYHIN1;PYHIN1	0.03	-0.22	UCEC
chr7	752456	+	cg13577076	PRKAR1B;PRKAR1B;PRKAR1B;PRKAR1B;PRKAR1B;PRKAR1B	0.01	-0.22	UCEC
chr18	31020511	-	cg05245861	C18orf34;C18orf34	0.02	-0.21	UCEC
chr21	31312328	-	cg21816539	GRIK1;GRIK1	0.01	-0.21	UCEC
chr8	73450181	+	cg20890210	KCNB2	0.04	-0.21	UCEC
chr10	128077323	+	cg13488201	ADAM12;ADAM12	0.04	-0.21	UCEC
chr18	74961424	+	cg12699371	GALR1	0.03	-0.21	UCEC
chr16	10277017	+	cg25047001	GRIN2A;GRIN2A;GRIN2A	0.05	-0.20	UCEC
chr6	62996697	+	cg02217159	KHDRBS2	0.01	-0.19	UCEC
chr11	131780946	-	cg11965370	NTM;NTM;NTM;NTM;NTM;NTM;NTM	0.01	-0.19	UCEC
chr3	21792434	+	cg18267381	ZNF385D;ZNF385D	0.05	-0.19	UCEC
chr1	38230769	+	cg12128017	EPHA10;EPHA10;EPHA10;EPHA10	0.04	-0.19	UCEC
chr13	27334259	+	cg22179082	GPR12	0.04	-0.19	UCEC
chr10	103603722	+	cg25123470	KCNIP2;KCNIP2;KCNIP2;KCNIP2;KCNIP2;KCNIP2	0.05	-0.19	UCEC
chrX	91034003	-	cg07474356	PCDH11X	0.02	-0.19	UCEC
chr10	101088930	-	cg01968793	CNNM1;CNNM1	0.01	-0.19	UCEC
chr18	74963364	-	cg00662556	GALR1	0.01	-0.19	UCEC
chr3	19190202	-	cg07657236	KCNH8;KCNH8	0.05	-0.18	UCEC
chr2	193059405	+	cg06856528	TMEFF2;TMEFF2	0.01	-0.18	UCEC
chr18	49868101	+	cg25602457	DCC	0.04	-0.18	UCEC
chr20	59826207	-	cg15534366	CDH4	0.00	-0.18	UCEC
chr1	24802	+	cg20855565	TRIM58	0.02	-0.18	UCEC

	0350						
chr5	151784441	+	cg03914397	NMUR2	0.04	-0.18	UCEC
chr19	44905343	-	cg09030119	ZNF285A	0.02	-0.18	UCEC
chr19	50831901	-	cg06572160	KCNC3	0.04	-0.18	UCEC
chr2	119606055	-	cg03266453	EN1	0.05	-0.18	UCEC
chr8	132054555	-	cg13912117		0.02	-0.17	UCEC
chr3	164914437	-	cg13619915	SLITRK3;SLITRK3	0.01	-0.17	UCEC
chr8	139164635	+	cg06276653	FAM135B	0.01	-0.17	UCEC
chr7	86273429	-	cg14724613	GRM3;GRM3	0.04	-0.17	UCEC
chr15	35046760	+	cg21053529	GJD2	0.02	-0.17	UCEC
chr17	80186273	-	cg14417329	SLC16A3;SLC16A3	0.00	-0.17	UCEC
chrX	55478180	+	cg18869368	MAGEH1	0.03	-0.17	UCEC
chr11	133402230	+	cg03923934	OPCML;OPCML	0.03	-0.17	UCEC
chr3	66550735	+	cg26131019	LRIG1	0.00	-0.17	UCEC
chr13	84456127	-	cg07104706	SLITRK1;SLITRK1	0.01	-0.17	UCEC
chr18	74964030	-	cg05896682	GALR1	0.02	-0.16	UCEC
chr3	62359420	+	cg06423920	FEZF2	0.01	-0.16	UCEC
chr9	104500729	-	cg08997253	GRIN3A;GRIN3A	0.00	-0.16	UCEC
chr1	3567214	-	cg19135761	WDR8	0.05	-0.16	UCEC
chr7	76038785	-	cg13594711	SRCRB4D;SRCRB4D;ZP3	0.02	-0.16	UCEC
chr19	44905882	-	cg26918645	ZNF285A	0.05	-0.16	UCEC
chr2	229046325	-	cg04072323	SPHKAP;SPHKAP;SPHKAP;SPHKAP	0.01	-0.16	UCEC
chr11	59855616	-	cg22197708	MS4A2;MS4A2	0.01	-0.16	UCEC
chr3	164914471	-	cg14717170	SLITRK3	0.02	-0.15	UCEC
chr11	2161586	-	cg25163476	INS-IGF2;IGF2;IGF2AS;IGF2;IGF2AS;IGF2	0.05	-0.15	UCEC
chr1	237205098	+	cg19764418	RYR2	0.01	-0.15	UCEC

chr1	206223241	+	cg18992688	AVPR1B	0.00	-0.15	UCEC
chr19	56510892	-	cg05023116	NLRP5	0.03	-0.15	UCEC
chr11	105481509	+	cg19343464	GRIA4;GRIA4;GRIA4;GRIA4;GRIA4	0.01	-0.15	UCEC
chr4	90757533	+	cg26578617	SNCA;SNCA;SNCA;SNCA	0.05	-0.15	UCEC
chrX	140991592	+	cg25651984	MAGEC1	0.01	-0.15	UCEC
chr4	156129725	-	cg27504805	NPY2R	0.04	-0.15	UCEC
chr2	110373002	+	cg03552103	SEPT10;SEPT10;ANKRD57	0.01	-0.15	UCEC
chr9	104501030	+	cg18794577	GRIN3A	0.01	-0.14	UCEC
chr1	236850232	-	cg21376883	ACTN2	0.04	-0.14	UCEC
chr12	5153288	+	cg20792062	KCNA5;KCNA5	0.01	-0.14	UCEC
chr10	26223100	+	cg08441170	MYO3A;MYO3A	0.00	-0.14	UCEC
chr9	122132261	+	cg25935911	DBC1	0.01	-0.14	UCEC
chr10	50817306	+	cg18592174	SLC18A3;CHAT	0.02	-0.14	UCEC
chr13	70682324	-	cg20523861	KLHL1;KLHL1;ATXN8OS	0.03	-0.14	UCEC
chr1	35220814	-	cg01333788	GJB5	0.00	-0.14	UCEC
chr18	44336399	-	cg19751300	ST8SIA5	0.04	-0.13	UCEC
chr19	41195910	+	cg08450982	NUMBL	0.02	-0.13	UCEC
chr19	31770476	-	cg08458170	TSHZ3	0.01	-0.13	UCEC
chr10	129691429	+	cg23817637	CLRN3	0.02	-0.13	UCEC
chr17	7906098	-	cg25465406	GUCY2D	0.05	-0.13	UCEC
chrX	85303309	-	cg25488547	CHM;CHM	0.02	-0.13	UCEC
chr11	113346327	+	cg12758687	DRD2;DRD2	0.05	-0.13	UCEC
chr6	78173250	-	cg08614481	HTR1B	0.02	-0.13	UCEC
chr7	3083338	+	cg26937500	CARD11;CARD11	0.01	-0.12	UCEC
chr1	66258441	-	cg26832142	PDE4B;PDE4B	0.00	-0.12	UCEC
chr4	147559579	-	cg13262687	POU4F2	0.03	-0.12	UCEC
chr7	130131258	+	cg13917504	MEST;MEST;MEST;MESTIT1;	0.03	-0.12	UCEC

				MEST			
chr3	4509268	-	cg18847227	SUMF1;SUMF1;SUMF1	0.02	-0.12	UCEC
chr1	37499309	+	cg06722633	GRIK3	0.04	-0.12	UCEC
chr18	56887785	-	cg23357981	GRP;GRP;GRP	0.04	-0.12	UCEC
chrX	144899412	-	cg27584469	SLITRK2;SLITRK2;SLITRK2;SLITRK2;SLITRK2;SLITRK2;SLITRK2;SLITRK2	0.02	-0.12	UCEC
chr19	57349815	+	cg19771589	PEG3;PEG3;ZIM2;PEG3;PEG3;ZIM2;ZIM2	0.02	-0.12	UCEC
chr20	30225517	-	cg27413508	COX4I2	0.02	-0.12	UCEC
chr6	133561932	-	cg20330472	EYA4;EYA4;EYA4	0.00	-0.12	UCEC
chr8	32405427	-	cg19162158	NRG1;NRG1;NRG1;NRG1;NRG1;NRG1;NRG1;NRG1;NRG1;NRG1;NRG1;NRG1	0.02	-0.11	UCEC
chr2	46523461	-	cg17518825	EPAS1	0.00	-0.11	UCEC
chr12	4554295	-	cg01731341	FGF6	0.03	-0.11	UCEC
chr5	1444878	+	cg26205131	SLC6A3	0.01	-0.11	UCEC
chr7	49813763	-	cg00333226	VWC2	0.05	-0.11	UCEC
chr10	26223310	-	cg23771603	MYO3A	0.01	-0.11	UCEC
chr5	44389282	+	cg20387341	FGF10	0.04	-0.11	UCEC
chrX	75647859	-	cg04544498	MAGEE1	0.02	-0.11	UCEC
chr17	39867328	+	cg12229387	GAST	0.00	-0.11	UCEC
chr2	73520043	-	cg13481359	EGR4	0.03	-0.11	UCEC
chr2	239009036	-	cg09039163	ESPNL;ESPNL	0.00	-0.10	UCEC
chr14	24804022	+	cg12265829	ADCY4	0.02	-0.10	UCEC
chr6	111302729	-	cg05213296	RPF2	0.01	-0.10	UCEC
chr19	4280054	-	cg18581445	SHD;SHD	0.01	-0.10	UCEC
chr16	71264290	-	cg20977864	HYDIN;HYDIN	0.05	-0.10	UCEC
chr9	130700866	-	cg23006204	DPM2	0.03	-0.10	UCEC

chr11	2017362	+	cg22172494	H19	0.01	0.10	UCEC
chr17	67137953	-	cg22081096	ABCA6;ABCA6	0.02	0.10	UCEC
chr9	130968072	+	cg05826823	CIZ1;DNM1;DNM1;CIZ1	0.02	0.11	UCEC
chr5	851101	+	cg18429742	ZDHHC11	0.01	0.11	UCEC
chr11	2018724	+	cg07342901	H19;MIR675	0.02	0.11	UCEC
chr4	26493496	-	cg04353483	CCKAR	0.04	0.11	UCEC
chr9	139939792	+	cg26581729	NPDC1	0.05	0.11	UCEC
chr6	152958899	-	cg27316956	SYNE1;SYNE1	0.00	0.11	UCEC
chr17	42092431	+	cg12259256	TMEM101	0.02	0.11	UCEC
chr22	37216067	+	cg02978737	PVALB	0.01	0.12	UCEC
chr11	2017664	-	cg25852472	H19	0.02	0.12	UCEC
chr22	38201690	+	cg07141002	H1F0	0.05	0.12	UCEC
chr2	220283175	+	cg18182399	DES;DES	0.01	0.13	UCEC
chr17	34345136	+	cg14916288	CCL23;CCL23	0.02	0.13	UCEC
chr11	8060060	-	cg15480475	TUB	0.01	0.13	UCEC
chr12	3861567	+	cg17904739	EFCAB4B;EFCAB4B;EFCAB4B	0.01	0.13	UCEC
chr8	118146418	-	cg23338195	SLC30A8	0.03	0.16	UCEC
chr21	33957290	+	cg20857253	TCP10L	0.03	0.17	UCEC
chr11	2017483	-	cg26808784	H19	0.00	0.19	UCEC
chrX	2882183	+	cg22376897	ARSE	0.00	0.24	UCEC
chrX	2882333	+	cg11964613	ARSE;ARSE	0.00	0.25	UCEC
chr3	123167276	-	cg13878010	ADCY5	0.01	0.29	UCEC
chr3	66550735	+	cg26131019	LRIG1	5.74E-14	-0.23	CESC
chr2	46523461	-	cg17518825	EPAS1	0.005	-0.15	CESC

Chr: chromosome of probe-associated gene
Pos: Genomic base position
Strand: + or – strand identiy
Name: DNA methylation probe name

UCSC RefGene Name
adj.P.Val: P value adjusted for multiple hypothesis testing with BH method
delta Beta: Difference in mean beta methylation level (EA – AA)
Tumor: CESC = cervix, UCEC = uterine endometrial carcinoma, OV = ovary, BRCA = breast
