# Supplementary material for: Race-associated Molecular Changes in Gynecologic Malignancies
Source: Cancer Res Commun. 2022 Feb 17;2(2):99–109. doi: 10.1158/2767-9764.CRC-21-0018 (PMC9390975; doi:10.1158/2767-9764.CRC-21-0018)
Supplement: Supplemental Table 7 — Differentially methylated probes identified independently in epigenetic studies of environment [file crc-21-0018-s07.pdf]

Supplemental Table 7

Name	Relation to Island	UCSC RefGene Name	UCSC RefGene Accession	UCSC RefGene Group	Publication
cg04601137	N_Shore	ADAMTSL5	NM_213604	5'UTR	*Joubert et al 2016*
cg17826679	Island	SLC44A2;SLC44A2	NM_020428;NM_001145056	TSS200;Body	*Joubert et al 2016*
cg13626881	OpenSea				*Joubert et al 2016*
cg23006204	S_Shore	DPM2	NM_003863	TSS200	*Joubert et al 2016*
cg26799474	OpenSea	CASP8;CASP8;CASP8;CASP8	NM_001080124;NM_033355;NM_033358;NM_001228	5'UTR;5'UTR;5'UTR;5'UTR	*Joubert et al 2016*
cg06144905	OpenSea	PIPOX	NM_016518	TSS200	*Joubert et al 2016*
cg05232889	OpenSea	FOXP2;FOXP2;FOXP2	NM_148898;NM_148899;NM_014491	5'UTR;5'UTR;5'UTR	*Jacobsen et al 2012*
cg27035169	OpenSea	SLC7A8	NM_012244	TSS1500	*Joubert et al 2016*
cg05331214	OpenSea	SCN7A	NM_002976	TSS1500	*Joehanes et al 2016*
cg15873301	N_Shore	SYN2;SYN2	NM_133625;NM_003178	TSS1500;TSS1500	*Galanter et al 2017*
cg08124030	OpenSea	TM4SF1	NM_014220	1stExon	*Jacobsen et al 2012*

Name: DNA methylation probe name
Relations to Island: position of methylation probe relative to nearest island
UCSC RefGene Name
UCSC RefGene Accession number
UCSC RefGeneGroup: Assigned DNA loci per UCSC designation
Publicaton: Publication associating probe with environmental stimuli
