# Supplementary material for: Race-associated Molecular Changes in Gynecologic Malignancies
Source: Cancer Res Commun. 2022 Feb 17;2(2):99–109. doi: 10.1158/2767-9764.CRC-21-0018 (PMC9390975; doi:10.1158/2767-9764.CRC-21-0018)
Supplement: Supplemental Figure S1 — Detailed patient racial assignments by tumor type and additional pathway analysis [file crc-21-0018-s08.pdf]

Figure S1

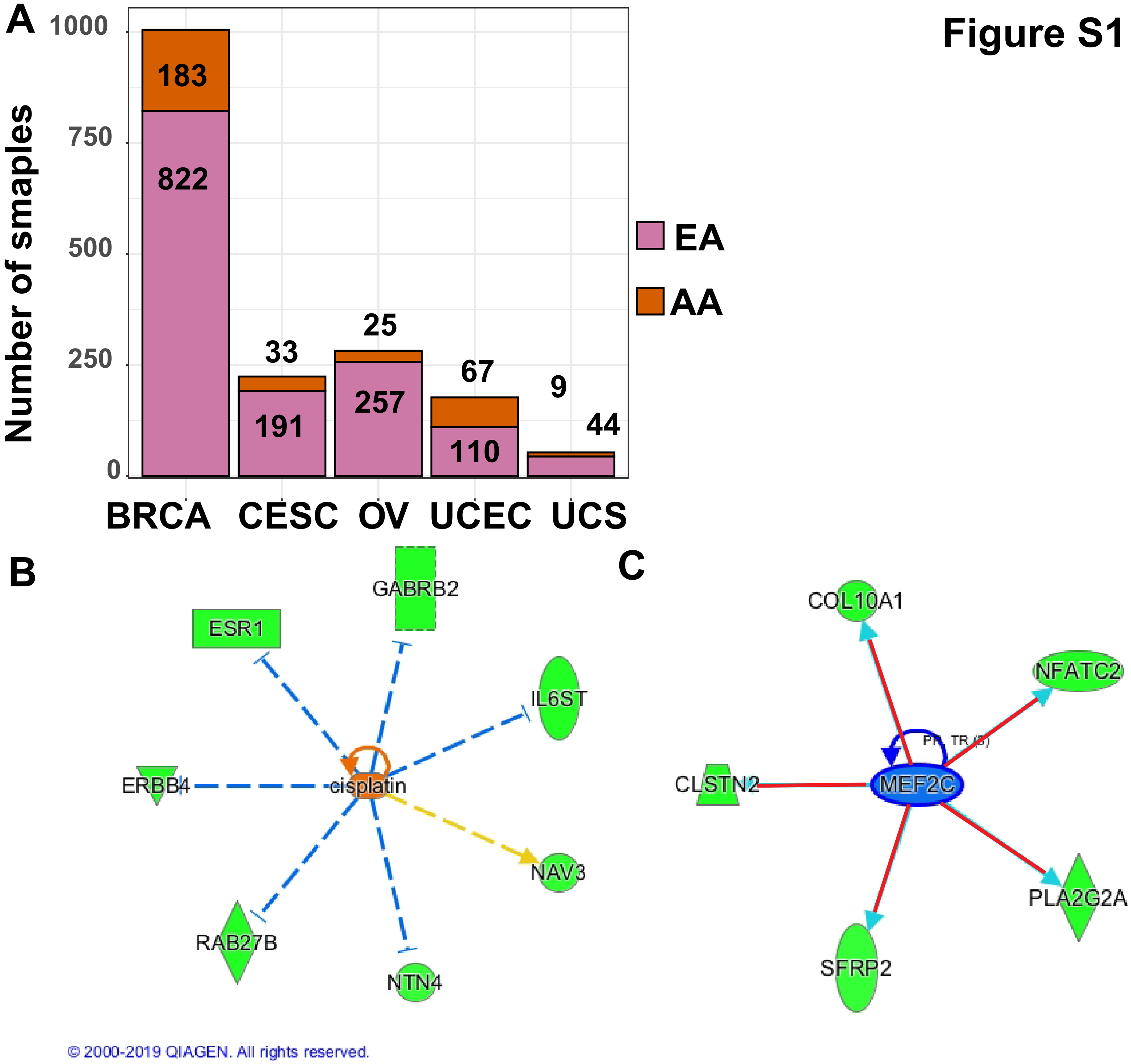

A. Numbers of African American (AA) and European American (EA) patients with each cancer type included in this analysis. BRCA, breast cancer; CESC, cervical cancer; OV, ovarian cancer; UCEC, uterine corpus endometrial cancer; and UCS, uterine carcinosarcoma.
B,C. Ingenuity Pathway Analyses of transcriptional network changes in the cisplatin- and MEFC2-associated expression pathways in AA vs. EA tumors. Relative changes in gene expression are depicted from lower (green) to higher (red) expression in AA samples. Database-predicted activator relationships are orange; predicted inhibitor relationships are blue, and red indicates unpredicted effects.
