# Supplementary material for: Race-associated Molecular Changes in Gynecologic Malignancies
Source: Cancer Res Commun. 2022 Feb 17;2(2):99–109. doi: 10.1158/2767-9764.CRC-21-0018 (PMC9390975; doi:10.1158/2767-9764.CRC-21-0018)
Supplement: Supplemental Figure S2 — Analysis of mRNA expression by tumor type [file crc-21-0018-s09.pdf]

**A**

## Ovary

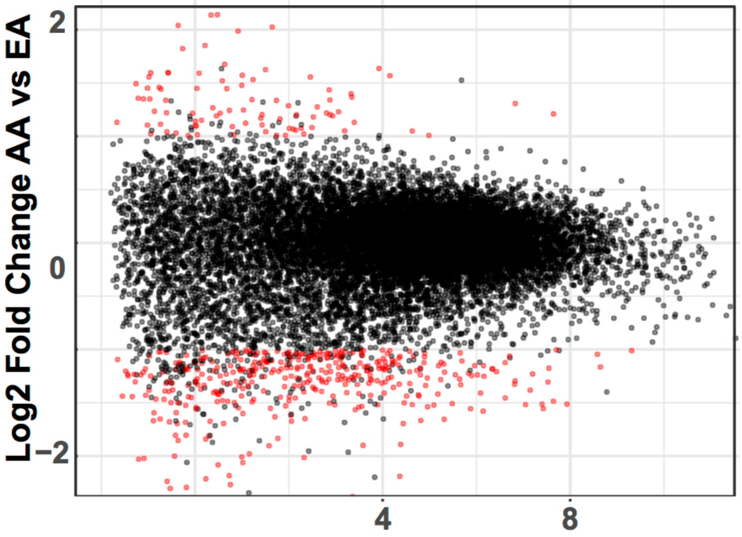


| Gene Set | NES | FDR q-val |
|---|---|---|
| Microtubule bundle formation | 3.1 | < 0.0001 |
| Cilium movement | 2.9 | < 0.0001 |
| Extracellular transport | 2.7 | < 0.0001 |
| DNA damage response via ATR | 2.15 | 0.002 |
| RNA export from nucleus | 2.05 | 0.01 |
| Regulation of leukocyte cell adhesion | -2.6 | < 0.0001 |
| Immune cell migration | -2.5 | < 0.0001 |
| Inflammatory response | -2.5 | < 0.0001 |

**B**

## Breast

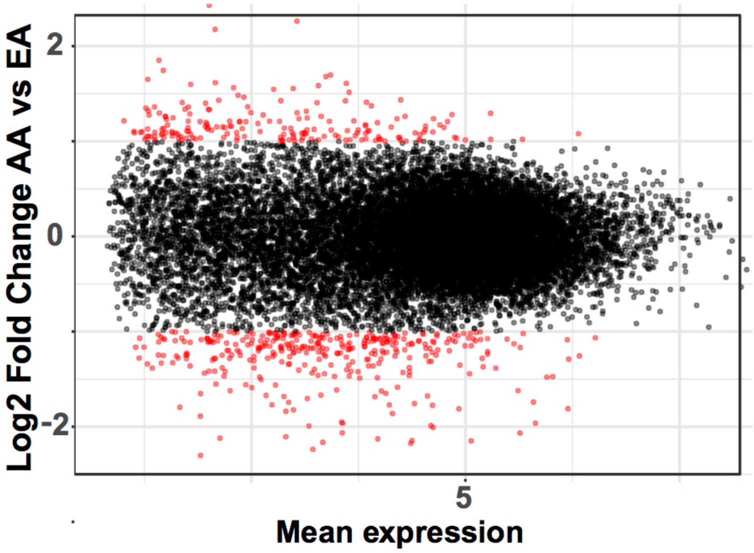


| Gene Set | NES | FDR q-val |
|---|---|---|
| DNA replication | 2.9 | < 0.0001 |
| DNA Methylation | 2.6 | < 0.0001 |
| Telomere maintenance | 2.5 | < 0.0001 |
| Cell cycle checkpoint | 2.15 | < 0.0001 |
| mir1305, mir944 | -2.9 | < 0.0001 |
| Plasma membrane adhesion | -2.7 | < 0.0001 |

**C**

## Uterine endometrial carcinoma

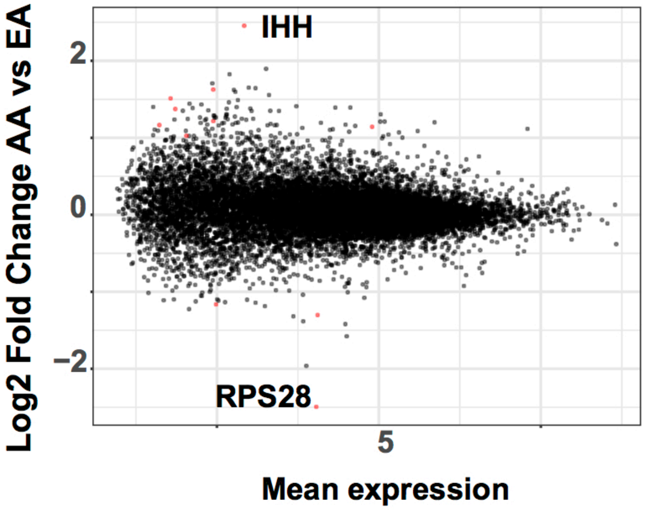


## Cervix

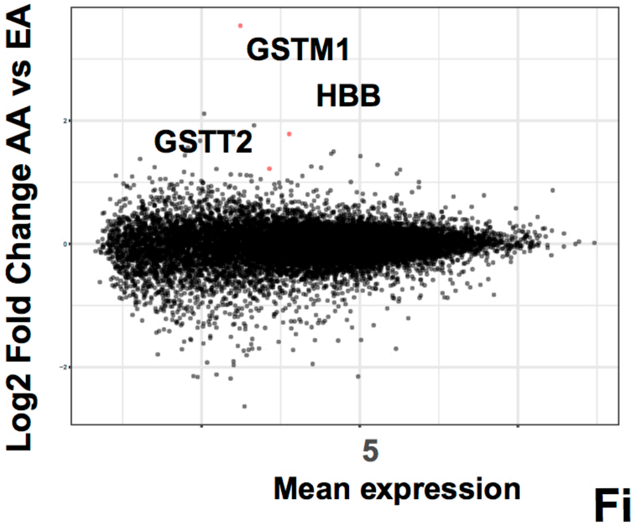


**Figure S2**

**Supplemental figure 2:** Individual tumor type transcriptional expression analysis.
Plot of all identified transcript mean reads per kilobase of transcript per million mapped reads (RPKM) across all samples vs. log transformed fold change in expression levels in AA vs. EA ($Log_2$ EA/AA). Significant transcripts were defined as those with two-fold expression change in AA vs EA samples and multiple hypothesis testing adjusted *P* values less than 0.05. Significant transcripts are highlighted in red. Names of the most-altered transcripts are noted on the plot.
Results of gene set enrichment analysis of transcriptional changes in individual tumors from AA compared to EA patients. For ovary (**A**) and breast (**B**) significantly altered gene sets identified via Gene Set Enrichment Analysis with normalized enrichment scores (NESs) are depicted. Gene sets up regulated in AA tumors are in red and gene sets down regulated in AA tumors are in blue. For uterine endometrial carcinoma and cervix small numbers of significantly altered genes precluded pathway analysis. The names of the top significant altered genes are depicted for uterus and cervix.
