# Supplementary material for: Race-associated Molecular Changes in Gynecologic Malignancies
Source: Cancer Res Commun. 2022 Feb 17;2(2):99–109. doi: 10.1158/2767-9764.CRC-21-0018 (PMC9390975; doi:10.1158/2767-9764.CRC-21-0018)
Supplement: Supplemental Figure S3 — Expression levels of RNA-processing pathway members [file crc-21-0018-s10.pdf]

Figure S3

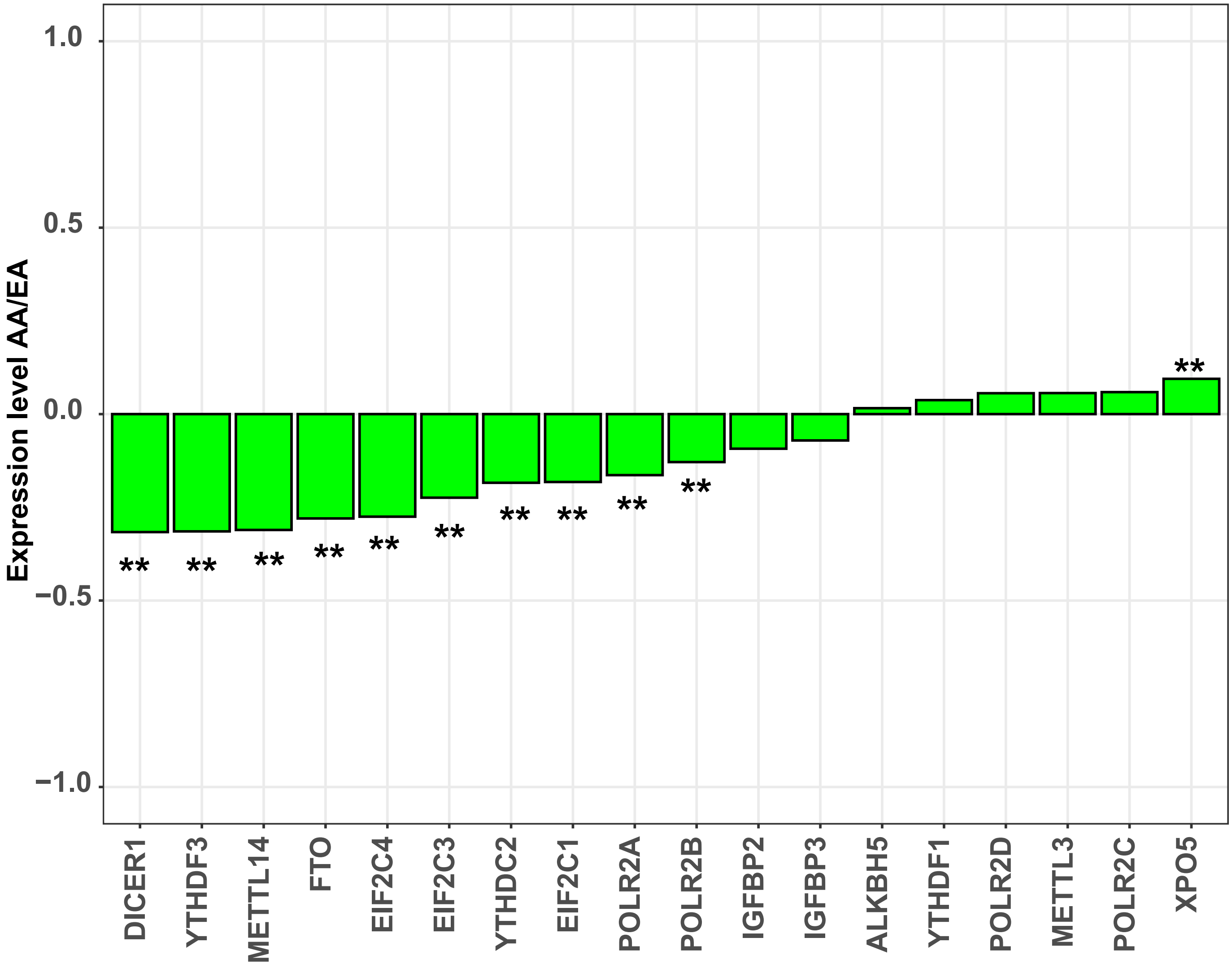

Log2 transformed expression levels (RPKM) of RNA processing pathway members in African American (AA) vs. European American (EA) samples AA/EA). Transcripts with lower values in AA samples compared to EA samples have a negative log fold change value. Statistically significant t tests with adjusted P values less than 0.01 are indicated with double asterisks.
