# Supplementary material for: Race-associated Molecular Changes in Gynecologic Malignancies
Source: Cancer Res Commun. 2022 Feb 17;2(2):99–109. doi: 10.1158/2767-9764.CRC-21-0018 (PMC9390975; doi:10.1158/2767-9764.CRC-21-0018)
Supplement: Supplemental Figure S4 — Analysis of methylation changes by tumor type [file crc-21-0018-s11.pdf]

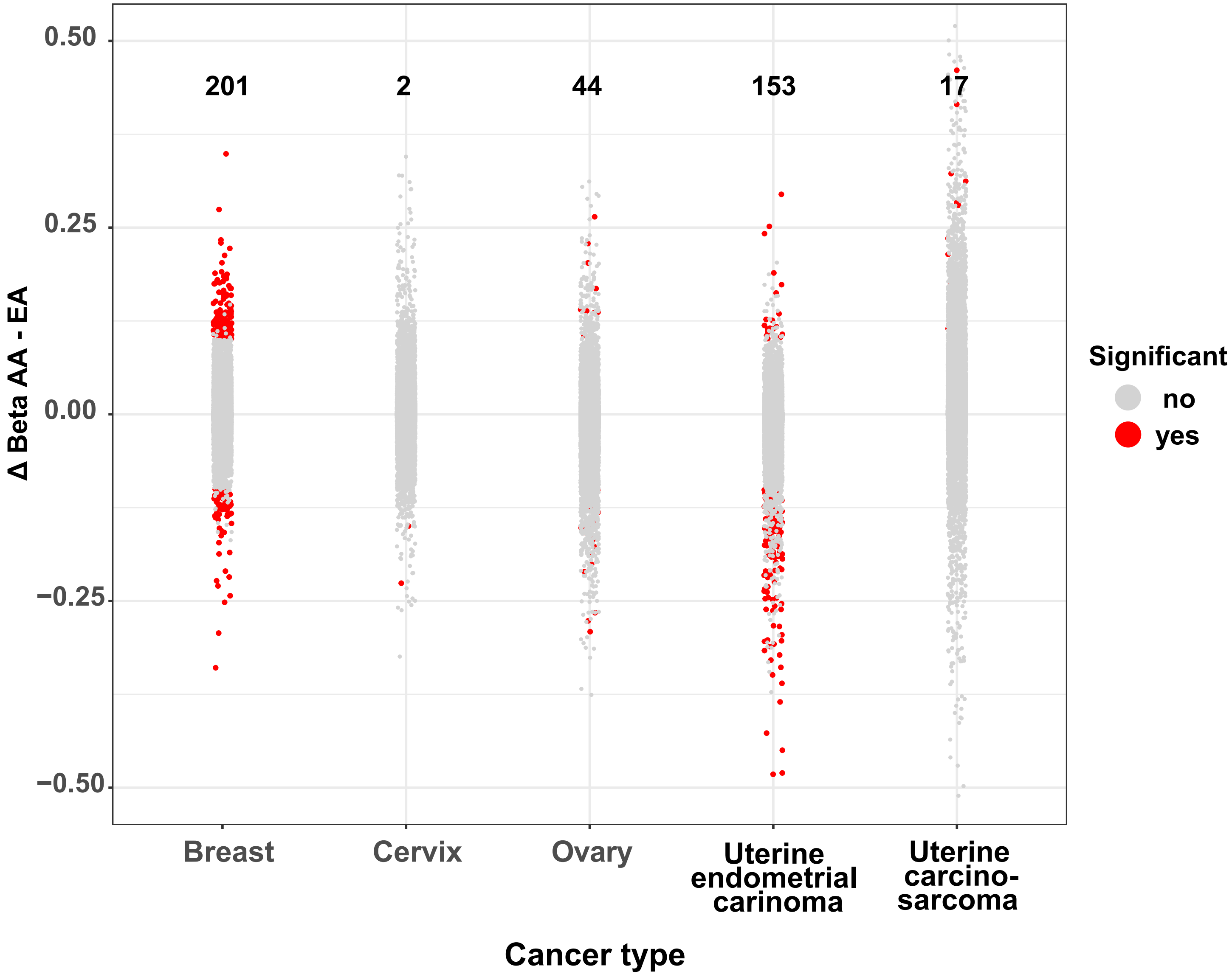

Tumor type-specific analysis of methylation changes in African American (AA) vs. European American (EA) individuals. The change in beta values between AA and EA tumors at each probe is shown on the y-axis. Significant probes are considered those with t test multiple hypothesis testing adjusted P values less than 0.05 and changes in beta values greater than 0.1. Significant probes are highlighted in red to indicate their significance. Numbers of significant altered probes are depicted above the dot plot for each tumor type.

Figure S4
