# Supplementary material for: Race-associated Molecular Changes in Gynecologic Malignancies
Source: Cancer Res Commun. 2022 Feb 17;2(2):99–109. doi: 10.1158/2767-9764.CRC-21-0018 (PMC9390975; doi:10.1158/2767-9764.CRC-21-0018)
Supplement: Supplemental Figure S5 — Analysis of differentially methylated probe locations [file crc-21-0018-s12.pdf]

Figure S5

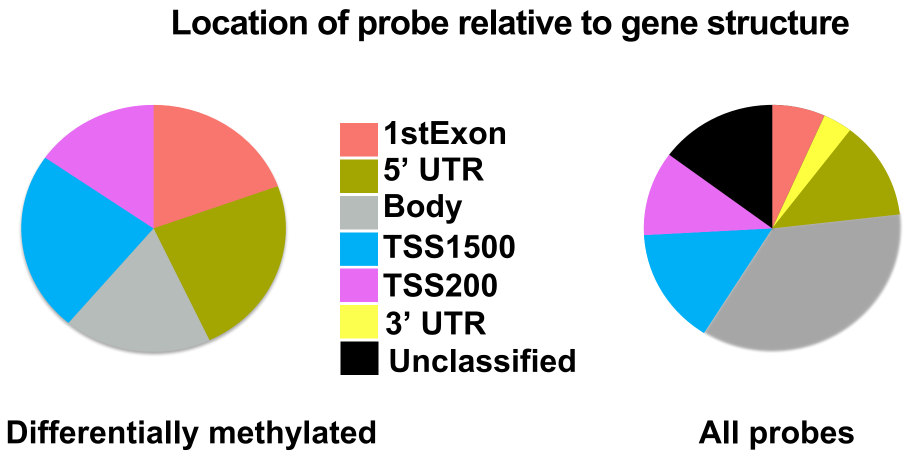

Pie charts showing location of probes with significant methylation changes in African American (AA) samples and locations of all probes relative to gene structure. UTR: untranslated region; TSS: transcription start site. TSS1500: 200 to − 1500 nt upstream of TSS. TSS200 from TSS to − 200 nt upstream of TSS. Relative to all probes this distribution is significantly different via Chi square test, p-value 1.5 X 10-12.
